# Environmental occurrence, exposure, toxicity, and transformation of benzothiazoles: A review

**DOI:** 10.1016/j.eehl.2026.100243

**Published:** 2026-04-24

**Authors:** Junʼan Bao, Sheng Wei, Ting Xu, Miao Cao, Huan Wang, Yiqun Song, Taoqin Chen, Daqiang Yin

**Affiliations:** a Key Laboratory of Yangtze River Water Environment, Ministry of Education, College of Environmental Science and Engineering, Tongji University, Shanghai 200092, China; b Postdoctoral Research Station of Environmental Science and Engineering, Tongji University, Shanghai 200092, China; c Shanghai Institute of Pollution Control and Ecological Security, Shanghai 200092, China; d Shanghai Center for Energy-saving and Emission Reduction, Shanghai 200232, China

**Keywords:** Benzothiazoles, Environmental occurrence, Human exposure, Toxicity, Toxic target, Environmental transformation

## Abstract

Benzothiazoles (BTs) are a class of aromatic heterocyclic compounds consisting of a benzene ring fused with a 1,3-thiazole ring. They have been widely used as vulcanization accelerators, fungicides, corrosion inhibitors, herbicides, anti-cancer drugs, etc., leading to their ubiquitous presence in various environmental media. Numerous studies have demonstrated that BTs exhibit multiple toxic effects. In this review, we systematically summarized research findings on the environmental occurrence, exposure, toxicity, and transformation of BTs. Benzothiazole (BTH) and 2-hydroxy-benzothiazole (2-OH-BTH) are the predominant BTs, with the highest detection frequencies and concentrations in environmental media and human biological samples. In some special cases, inhalation is estimated to be the primary exposure pathway, compared with ingestion and dermal contact. BTs have been reported to possess multiple toxic effects, including neurotoxicity, developmental and reproductive toxicity, endocrine-disrupting effects, and immunotoxicity. By integrating network toxicology analysis and reported experimental results, we highlighted that the aryl hydrocarbon receptor could be the key toxic target of BTs. BTs can further transform into diverse derivatives in wastewater treatment systems and the natural environment, with BTH and 2-OH-BTH being the main transformation products. The TP-Transformer model predicted that some BT-derived pharmaceuticals can be transformed into 2-amino-benzothiazole (2-ABTH), indicating that they are potential sources of environmental pollution. The predicted toxicity of most transformation products was reduced, while chlorinated derivatives exhibited increased toxic potential. Finally, we proposed BT-derived pharmaceuticals as a potential neglected source of BTs, and more attention should be given to the precise identification of transformation products and understanding the toxic mechanisms.

## Highlights

• BTH and 2-OH-BTH are the most abundant benzothiazoles (BTs) in both environment and human.
• Aryl hydrocarbon receptor was identified as a common toxic target for BTs.
• BTH and 2-OH-BTH are the main transformation products during wastewater treatment and natural light radiation.
• Chlorinated derivatives exhibited enhanced toxicity, whereas most other transformation products reduced.
• BT-derived drugs are an important but neglected source of BTs in the environment.

## Graphical abstract

**Figure:**
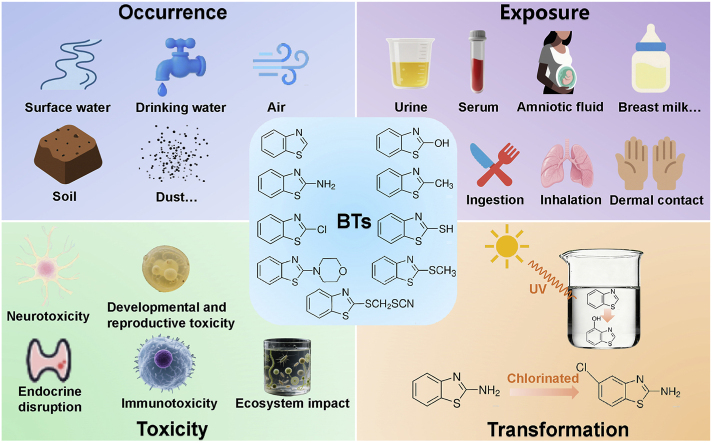

## 1 Introduction

Tire wear particles (TWPs) have been raising public concerns since the identification of the quinone derivative of N-(1,3-dimethylbutyl)-N′-phenyl-p-phenylenediamine (6PPD), a widely used antioxidant in tires, as the principal culprit for the acute mortality in Pacific Northwest coho salmon (*Oncorhynchus kisutch*) during their spawning migration [1]. TWPs are comprised of a variety of chemicals, including 6PPD (antioxidant), benzothiazoles (BTs, vulcanization accelerators), benzotriazoles (BZTs, ultraviolet light stabilizers or corrosion inhibitors), etc. These additive chemicals will eventually enter environmental media through multiple pathways (e.g., urban runoff, seepage, wastewater effluent, etc.). BTs have been reported to be the most abundant chemicals in the TWP leachates. In the TWPs leachate extracted with distilled water, the concentration of BTs reached 24–91 μg/L [2], while the TWPs leachate extracted with manually prepared freshwater and seawater contained an even higher concentration of BTs (freshwater: 2313 μg/L; seawater: 1460 μg/L) [3]. The substantial release of BTs from TWPs into the environment has made them emerging contaminants of particular concern among tire additive chemicals.

BTs are a class of aromatic heterocyclic compounds formed by the combination of a 1,3-thiazole ring and a benzene ring. Aside from being used as vulcanization accelerators, the superior biological activity of the benzothiazole nucleus leads to the wide application of BTs as fungicides [4], corrosion inhibitors [5], herbicides [6], and anti-cancer drugs [7]. Multiple BTs (benzothiazole and its 2-substituted derivatives) have been widely detected in a variety of environmental media, including surface water [8], drinking water [9], groundwater [10], wastewater [11], air [12], dust [13], soil [14], sediment [15], sludge [16], etc. As a result, humans are inevitably exposed to these chemicals through ingestion, inhalation, and dermal contact. The existence of BTs in a lot of human samples has also been verified (urine [17], amniotic fluid [18], adipose tissue [19], serum [20], semen [21], etc.). More alarmingly, numerous epidemiological studies and animal model experiments have revealed that BTs exhibit multi-target toxic effects, including neurotoxicity [22], endocrine disruption [23], immunotoxicity [24], as well as developmental and reproductive toxicity [25,26]. Although existing studies have shed some light on the toxic effects of BTs, knowledge regarding the toxic targets and underlying mechanisms remains limited. In addition, BTs are prone to transformation in the environment [27], and research regarding the toxicity of these transformation products is even more scarce.

Due to their widespread occurrence and associated toxic effects, BTs have attracted increasing attention. In 2018, Liao et al. reviewed the environmental occurrence, fate, exposure, and toxicity of BTs [28]. This review provides updated information on the occurrence of BTs across various environmental media, comprehensively summarizes the exposure pathways as well as human exposure levels, and offers a more detailed discussion of their toxic effects. We further integrate data from open-access toxicology databases and computational toxicological models to explore the potential toxic targets of BTs and evaluate the toxicity of their transformation products. Based on the comprehensive summary of the findings, we identify current research gaps and propose directions for future investigation.

## 2 Methods

### 2.1 Literature search strategy

**Fig. 1:**
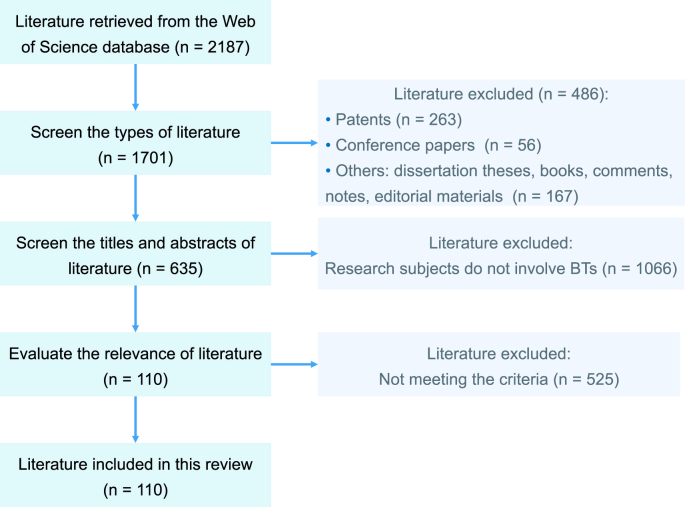
The framework for the literature screening strategy used in this review.

In this review, relevant literature published up to December 2024 was retrieved from the Web of Science database. The following keywords were used individually or in combination during the search: “benzothiazoles”, “occurrence”, “exposure”, “toxicity”, “transformation”, and “BTs”. The search was restricted to English-language publications, yielding a total of 2187 articles. Firstly, by screening the publication types of the literature, patents, conference papers, and other (dissertation theses, books, comments, notes, and editorial materials) were excluded, resulting in 1701 articles. Then, by screening the titles and abstracts of the literature, articles whose research subjects did not involve BTs were excluded, leaving 635 articles. Finally, articles that met the following criteria were included in the scope of this review: (1) Study one or more BTs within the scope of this review; (2) At least one research topic of this review should be involved, including “environmental occurrence”, “exposure”, “toxicity”, and “transformation”; (3) The methods were described clearly and the data supported the conclusions. A total of 110 articles were ultimately included in this review. The detailed literature screening process is illustrated in Fig. 1.

### 2.2 Exploration of potential toxic targets of BTs

Public toxicology databases, including TargetNet, SwissTargetPrediction, ChEMBL, and Tox21, were utilized to explore the potential toxic targets of several commonly detected BTs in the environmental media, including benzothiazole (BTH; CAS: 95-16-9), 2-mercapto-benzothiazole (MBT; CAS: 149-30-4), 2-hydroxy-benzothiazole (2-OH-BTH; CAS: 934-34-9), 2-methylthio-benzothiazole (2-Me-S-BTH; CAS: 615-22-5), 2-amino-benzothiazole (2-ABTH; CAS: 136-95-8), 2-chlorobenzothiazole (2-Cl-BTH; CAS: 615-20-3), 2-methyl-benzothiazole (2-Me-BTH; CAS: 120-75-2), 2-thiocyanomethylthio-benzothiazole (2-SCN-Me-S-BTH; CAS: 21564-17-0), and 2-(4-Morpholinyl)benzothiazole (2-Mo-BTH; CAS: 4225-26-7). Firstly, the SMILES (Simplified molecular input line entry system) of individual BTs were retrieved from the PubChem database. Subsequently, the potential toxic targets were predicted using their respective SMILES for TargetNet and SwissTargetPrediction or compound names for ChEMBL. In all databases, the species was specified as “Homo sapiens”. Additionally, for TargetNet, the Area Under Curve (AUC) threshold was set to 0.9, and the predicted targets with a “Probability” value of 1 were finally adopted. For results obtained from SwissTargetPrediction and ChEMBL, all positive targets were directly included. Finally, the targets obtained from the three databases were consolidated, and duplicates were removed, resulting in a final set of predicted positive targets for each compound. Additionally, the Tox21 toxicity database was employed to screen for experimentally positive targets by selecting only those entries with an “Activity” designation of “Active”. Ultimately, by integrating both the predicted positive targets and experimentally confirmed active targets, shared potential toxic targets of BTs were screened.

### 2.3 Computational prediction of transformation products of BTs and their toxicity

We randomly selected seven commercial or ongoing clinical trials BT-derived pharmaceuticals with different therapeutic applications to explore their potential transformation products, including Riluzole (for the treatment of amyotrophic lateral sclerosis, CAS: 1744-22-5), Frentizole (antiviral, CAS: 26130-02-9), Pramipexole (for the treatment of Parkinsonʼs disease, CAS: 104632-26-0), Ethoxzolamide (for the treatment of glaucoma, CAS: 452-35-7), Zopolrestat (for the treatment of diabetes, CAS: 110703-94-1), 2-(4-aminophenyl)benzothiazole (antitumor, in early-phase clinical trial, CAS: 6278-73-5), and MB710 (antitumor, in early-phase clinical trial, CAS: 2230044-57-0). The prediction of transformation products was conducted using the TP-Transformer model, developed by Dai et al. [29], which is primarily designed for predicting transformation products generated during the chemical oxidation of organic pollutants. The SMILES of compounds were input into the model under the following reaction conditions: reacted with HO**·**, no energy input, and the influence of pH value was not considered. The transformation products were then generated as output. In accordance with the model guidelines, only the top five transformation products with the highest predicted probabilities (top 1 to top 5) were retained from the ten initially predicted. The toxicity (both acute and chronic) of BTsʼ transformation products was then predicted using the widely adopted Ecological Structure Activity Relationships (ECOSAR v2.2) model (https://www.epa.gov/tsca-screening-tools/ecological-structure-activity-relationships-ecosar-predictive-model). In brief, the CAS numbers or SMILES of the compounds were imported into the software for toxicity prediction. To ensure comparability across different compounds, all prediction results were consistently generated using the “Chemical Classes” option set to “Neutral Organics”.

## 3 Occurrence and human exposure

### 3.1 Environmental occurrence

#### 3.1.1 Surface water and groundwater

**Fig. 2:**
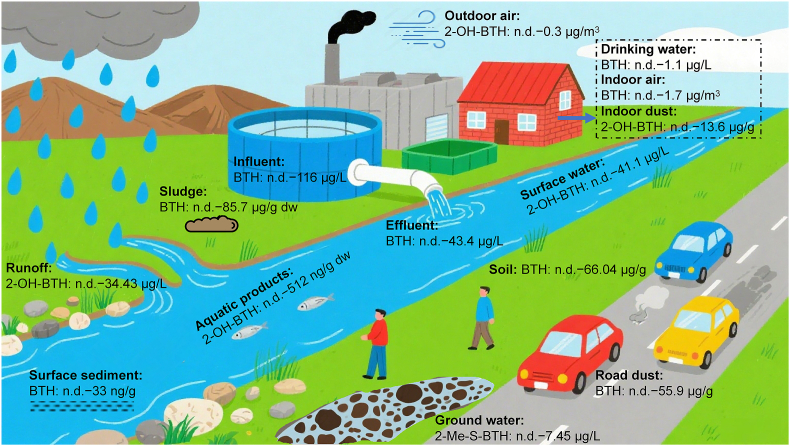
The predominant BTs and their detected concentrations in different environmental media. The chemical name listed under each environmental medium indicates the BT with the highest detection frequency and peak concentration. n.d., not detected.

We summarized the distribution of BTs in various environmental media (Table S1), and the predominant BTs in different environmental media are shown in Fig. 2. It should be noted that the predominant BTs proposed in Fig. 2 are mainly based on their high detection frequencies and concentrations in existing studies, which largely depend on the sensitivity of the analytical methods adopted and the actual range of chemicals detected in these studies.

Zhang et al. examined the levels of four BTs in surface water samples from the Pearl River Estuary, China. 2-OH-BTH was identified as the most predominant compound, with concentration ranges of 23.1−191 ng/L in the dry season and 34.7−1652 ng/L in the wet season, significantly higher than BTH, 2-ABTH, and 2-Me-S-BTH (dry season: <0.09 to 99.6 ng/L; wet season: 0.14−285 ng/L) [30]. In groundwater samples from Guangzhou, China, 2-Me-S-BTH reached a maximum concentration of 7446 ng/L, making it the most dominant BT compound detected [31]. BTs have also been detected at varying levels in surface water and groundwater from multiple countries, including Brazil [32], South Korea [33], the United States [34], and Denmark [35]. The concentrations of BTs in the urban surface water showed a significant increase after rainfall. It was reported that 2-OH-BTH, 2-ABTH, and 2-Me-S-BTH could rise to the peak concentration of 449, 77, and 274 ng/L, respectively, in a tributary of the Brisbane River, Australia, during heavy rainfall events [8]. Notably, 2-OH-BTH and 2-Me-S-BTH exhibited higher detection frequencies and concentrations than other BTs in surface water and groundwater, respectively, establishing them as the dominant BTs in these environmental compartments.

#### 3.1.2 Drinking water

The widespread occurrence of BTs in surface water and groundwater further affects drinking water supplies. Analysis of 86 drinking water samples from 51 Chinese cities revealed the prevalent presence of three target BTs (BTH, 2-OH-BTH, and 2-ABTH), with 2-Cl-BTH being undetectable. BTH exhibited universal detection (100%) at concentrations of 32.5−1100 ng/L. 2-OH-BTH showed the next-highest detection frequency (59.3%), followed by 2-ABTH, which was detected in only 8.1% of the samples [9]. The Korean drinking water samples exhibited comparable BT detection profiles, but at substantially reduced concentrations (total concentration range: 18.7−149 ng/L) versus Chinese samples [33]. Among the various BTs detected in drinking water, BTH demonstrated predominant occurrence, establishing it as the primary compound of concern.

#### 3.1.3 Roadside soil

Urban roadside soil contained detectable levels of BTs, likely attributable to inputs from TWPs. In 47 roadside soil samples from the northeastern United States, BTH, 2-OH-BTH, and 2-Me-S-BTH showed high detection frequencies (98%−100%) and concentrations, with mean levels of 370, 56, and 51 ng/g dry weight (dw), respectively, while 2-ABTH exhibited significantly lower contamination at just 4.5 ng/g dw [14]. Another study investigated BTs in roadside soil from Tianjin, China. Among the five detected BTs (BTH, MBT, 2-OH-BTH, 2-ABTH, and 2-Me-S-BTH), BTH was the most abundant compound, reaching a maximum concentration of 66 μg/g, which was several to dozens of times higher than the other four BTs [36]. Mirroring patterns observed in drinking water, BTH emerged as the predominant BT species in soil as well.

#### 3.1.4 Air and dust

BTs have also been detected at measurable concentrations in both atmospheric and dust samples. In ambient air samples collected around an industrial area of Spain across different seasons, BTH was the most frequently detected (86%−100%) and abundant compound, with mean concentrations ranging from 624 pg/m^3^ (winter PM_10_) to 1217 pg/m^3^ (summer gas phase). The mean concentrations of 2-Cl-BTH, 2-OH-BTH, and 2-Me-S-BTH varied from not detected (n.d.) (2-OH-BTH; summer gas phase) to 382 pg/m^3^ (2-Me-S-BTH; summer gas phase), whereas 2-ABTH was only detected at trace levels (<method quantification limit) in summer PM_10_ samples [37]. Analysis of 158 indoor dust samples from four countries (USA, China, Japan, and South Korea) revealed 2-OH-BTH as the most ubiquitous and dominant compound, detected in all samples with concentrations ranging from 3.85 ng/g (China) to 13,600 ng/g (USA). 2-Me-S-BTH and 2-SCN-Me-S-BTH showed secondary prevalence, while 2-ABTH exhibited the lowest detection frequency and concentration levels [38]. Distinct dominant BTs were identified between ambient and indoor air: 2-OH-BTH prevailed in outdoor environments, whereas BTH dominated indoor air. In addition, 2-OH-BTH accounted for a substantially larger proportion in indoor dust.

### 3.2 Exposure pathways

Human exposure to BTs occurs primarily through three pathways: ingestion, inhalation, and dermal contact. In South Korea, the estimated daily intakes (EDIs) of BTH through drinking water consumption ranged from 0.851 ng per kg body weight (bw) per day [ng/(kg⋅d); median scenario, teenagers] to 4.53 ng/(kg⋅d) (worst scenario, toddlers) [33]. Based on BTsʼ occurrence data in mollusks from Chinaʼs Bohai Sea, Jia et al. [39] estimated the total EDIs of BTs by local populations at 76.1−124 ng/(kg⋅d). BTH accounted for the largest proportion [58.2−94.9 ng/(kg⋅d)], representing approximately 9-fold and 24-fold higher exposure levels than those of 2-SCN-Me-S-BTH [6.57−10.7 ng/(kg⋅d)] and 2-Me-BTH [2.36−3.85 ng/(kg⋅d)], respectively. Among the ten most consumed seafood species in Spainʼs Tarragona region, BTH and 2-Me-S-BTH were identified as the most abundant BTs compounds, with the maximum EDIs reaching 11 and 22 ng/(kg⋅d), respectively. Higher exposure levels were observed in elderly individuals and females compared to young adults and males [40]. Additionally, the average EDIs of BTH via leafy vegetable consumption were determined to be 52 and 12 ng/(person⋅d) for populations in Israel and Switzerland, respectively [41].

BTs adsorbed onto particulate matter (e.g., dust, PM_2.5_) could enter the human body through ingestion or inhalation. Children in the USA, South Korea, China, and Japan exhibited higher EDIs of BTs through indoor dust ingestion [0.520−4.221 ng/(kg⋅d)] compared to adults [0.104−0.911 ng/(kg⋅d)] [38]. For residents in Guangzhou, Shanghai, and Taiyuan, China, the mean EDIs of BTs via inhalation of outdoor PM_2.5_ were 113−192 ng/(kg⋅d) for children versus 39.4−66.9 ng/(kg⋅d) for adults, again demonstrating significantly higher exposure levels in pediatric populations [25].

Dermal contact with BT-containing materials in daily life represented another critical exposure pathway. Analysis of 79 clothing textile samples collected from Albany, New York, detected BTH, 2-Me-S-BTH, and 2-OH-BTH. Infant clothing with printed graphics and decorative portions, as well as socks containing certain synthetic fibers, were identified as primary contributors to dermal exposure [42]. A Europe-wide risk assessment study of rubber crumb from artificial turf revealed that human dermal exposure levels to BTH, 2-OH-BTH, and MBT ranged 0.0314−0.89, 0.0265−0.751, and 0.0632−1.79 μg/(kg⋅d), respectively [43,44].

### 3.3 Human exposure levels

The first report regarding the presence of BTs in humans dates back to 1985, when researchers detected over 150 chemicals, including BTH at a concentration of 10 ng/g, in thrombotic coronary plaques from autopsy specimens of cardiac patients [45]. The content of BTs in urine can usually serve as a biomarker for assessing internal exposure to environmental contaminants. A comprehensive analysis of 332 urine samples from seven countries identified BTH as the dominant compound, with detection frequencies of 24%–100% and geometric mean (GM) concentrations of 5.1–22.7 μg/L, accounting for 43% of total BTs on average. In contrast, 2-Mo-BTH, 2-OH-BTH, and 2-ABTH showed substantially lower detection rates (n.d.–19.4%) and GM concentrations (n.d.–7.5 μg/L) [46]. In urine samples collected from the Australian general population during 2012–2023, 2-OH-BTH emerged as the predominant compound, exhibiting a detection frequency of 76%–80% and a mean concentration of 12 μg/L—approximately three times that of BTH (4.3–4.7 μg/L). In contrast, 2-Me-S-BTH was detected at markedly lower frequencies (1.2%–6.0%) [17]. Nevertheless, in a recent analysis of 197 adult urine samples from Taizhou, China, 2-Me-S-BTH demonstrated a higher detection frequency (76%) with a GM concentration of 0.81 μg/L [47].

The detection of BTs was not consistently reported among different studies. In urine samples from 6 Italian volunteers, all of the target BTs (BTH, 2-Me-BTH, 2-Me-S-BTH, 2-ABTH, 2-OH-BTH, and MBT) were below the limit of detection (LOD) [48]. None of the BTs were detected in drinking water and other samples from the volunteersʼ residential environment, which might explain the negative detection results of BTs in urine samples. In addition, the limited sample size could be another possible reason for the negative results. Similar results were observed in urine samples from 20 volunteers in Taiwan, China. Neither BTH nor 2-Me-S-BTH was detected in any sample, while 2-OH-BTH was quantifiable in only 10% of female samples at trace levels (<limit of quantitation, LOQ) [49]. It should be noted that this study employed UHPLC-QTOF-MS for quantifying the concentration of BTs, resulting in the LOQ being around one order of magnitude higher than that achieved by UHPLC-QQQ-MS [48,50], which could contribute to the failure in detecting BTs in urine samples. Hence, particular attention should be paid to the analytical method when evaluating human exposure levels.

**Fig. 3:**
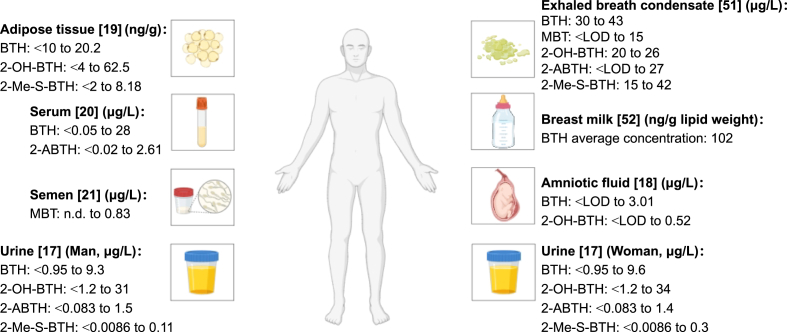
Distribution of BTs in different human matrices. The text in the figure shows the types and concentration ranges of BTs detected from different human matrices in these studies. LOD, limit of detection.

Besides urine, studies have also investigated BTsʼ occurrence in other human matrices, as shown in Fig. 3, including adipose tissue [19], serum [20], exhaled breath condensate [51], breast milk [52], semen [21], etc., with diverse BTs types and levels detected. More information on the occurrence of BTs in different human matrices is provided in Table S2. These findings demonstrated the wide distribution of BTs across human matrices.

## 4 Toxic effects

A systematic summary of reported toxicological endpoints and corresponding effect concentrations for BTs is presented in Table 1 [25,[53], [54], [55], [56], [57], [58], [59], [60], [61], [62], [63], [64], [65], [66], [67], [68], [69], [70], [71], [72], [73], [74], [75], [76]].

**Table 1:** Summary of BTs toxicity^a^.

Compound	Neurotoxicity		Developmental and reproductive toxicity		Endocrine disruption		Immunotoxicity		Acute toxicity		Others	
	End point	Concentration	End point	Concentration	End point	Concentration	End point	Concentration	End point	Concentration	End point	Concentration
BTH	Zebrafish larvae (semi-static renewal, 144 h), inhibited neuron development [54]	OEC: 10 μM	1) *Phaeodactylum tricornutum* (waterborne, 34 d), growth inhibition [69] 2) Water flea (static renewal, 7 d), growth inhibition [70]	1) LOEC: 5 mg/L; EC_50_: 41 mg/L 2) NOEC: 11.9 mg/L; EC_50_: 54.9 mg/L	1) *Xenopus laevis* tadpole thyroid gland, thyroxine (T4) release inhibition [63] 2) Porcine thyroid gland, TPO inhibition [63] 3) Recombinant yeast, activate ER [67]	1) IC_50_: 457 μM 2) IC_50_: 10,000 μM (40% inhibition) 3) EC_50_: 5.5 mg/L			1) Sheepshead minnow larvae (waterborne, 120 h), mortality [72] 2) Human carcinoma cell MGC-803, mortality [73] 3) Rat (oral), mortality [74] 4) Water flea (static nonrenewal, 48 h), mortality [70]	1) LC_50_: 41.9 mg/L 2) LC_50_: 160 mg/L 3) LD_50_: 380–900 mg/kg 4) LC_50_: 24.6 mg/L	1) Recombinant yeast, activate AhR [67] 2) Rainbow trout epithelial cell line RTgill-W1 (waterborne, 12 d), activate AhR [71] 3) Rainbow trout epithelial cell line RTL-W1 (waterborne, 1 d), activate AhR [71]	1) EC_50_: 10.2 mg/L 2) EC_50_: 76.2–106.9 mg/L 3) EC_50_: 20.3–31.6 mg/L
MBT	1) Zebrafish larvae (semi-static renewal, 144 h), inhibited neuron development [54] 2) Zebrafish larvae (static renewal, 120 h), eye injury [58]	1) OEC: 10 μM 2) OEC: 0.3–0.6 mg/L	1) Water flea (static renewal, 7 d), growth inhibition [70] 2) Chicken embryo (air sac injection, 11 d), malformation [60] 3) Rat (intraperitoneal, 21 d), malformation [62]	1) NOEC: 0.84 mg/L; EC_50_: 1.25 mg/L 2) ED_50_: 2 μM 3) OEC: 200 mg/kg/d	1) *Xenopus laevis* tadpole thyroid gland, thyroxine (T4) release inhibition [63] 2) *Xenopus laevis* larvae (flow-through, 7 d), TPO inhibition [64] 3) Porcine thyroid gland, TPO inhibition [63] 4) Zebrafish larvae (static renewal, 168 h), TPO inhibition [66] 5) Fathead minnow larvae (static renewal, 21 d), TPO inhibition [65]	1) IC_50_: 3 μM 2) LOEC: 18–174 μg/L; IC_50_: 1.92 mg/L 3) IC_50_: 12 μM 4) EC_50_: 3.2 mg/L 5) OEC: 1 mg/L	1) Mice (local lymph node assay, 3 d), sensitization [68] 2) Guinea pig (Buehler assay, 14 d), sensitization [68]	1) OEC: 4.2% 2) OEC: 5%–10%	1) *Salmonella typhimurium* TA1535/pSK1002 -S9, mortality [73] 2) Rat (oral), mortality [74] 3) Water flea (static nonrenewal, 48 h), mortality [70] 4) Zebrafish larvae (static nonrenewal, 120 h), mortality [58] 5) Zebrafish larvae (static renewal, 168 h), mortality [66] 6) Mice (intraperitoneal, 72 h), mortality [53] 7) Mice (oral, 72 h), mortality [53] 8) Rainbow trout (semi-static renewal, 96 h), mortality [75]	1) LC_50_: 19 mg/L 2) LD_50_: 100–7500 mg/kg 3) LC_50_: 4.19 mg/L 4) LC_50_: 1.51 mg/L 5) LC_50_: 4.2 mg/L 6) LD_50_: 437 mg/(kg⋅d) 7) LD_50_: 2000 mg/(kg⋅d) 8) LC_50_: 1.3–6.2 mg/L	1) Rainbow trout epithelial cell line RTgill-W1 (waterborne, 12 d), activate AhR [71] 2) Rainbow trout epithelial cell line RTL-W1 (waterborne, 1 d), activate AhR [71]	1) EC_50_: 16.7–18.5 mg/L 2) EC_50_: 63.7–104.6 mg/L
2-OH-BTH	Zebrafish larvae (semi-static renewal, 144 h), abnormal expression of genes related to neurodevelopment [54]	OEC: 10 μM	1) *Raphidocelis subcapitata* (waterborne, 34 d), growth inhibition [69] 2) Water flea (static renewal, 7 d), growth inhibition [70] 3) Zebrafish larvae (static renewal, 96 h), malformation [25]	1) EC_50_: 16 mg/L 2) NOEC: 2.74 mg/L; EC_50_: 8.31 mg/L 3) EC_50_: 287 μM	1) *Xenopus laevis* tadpole thyroid gland, thyroxine (T4) release inhibition [63] 2) Recombinant yeast, activate ER [67]	1) IC_50_: 100 μM 2) EC_50_: 6.346 mg/L			1) *Salmonella typhimurium* TA1535/pSK1002 +S9, mortality [73] 2) Water flea (static nonrenewal, 48 h), mortality [70] 3) Zebrafish larvae (static renewal, 96 h), mortality [25]	1) LC_50_: 24 mg/L 2) LC_50_: 15.1 mg/L 3) LC_50_: 358 μM	1) Recombinant yeast, activate AhR [67] 2) Rainbow trout epithelial cell line RTgill-W1 (waterborne, 12 d), activate AhR [71] 3) Rainbow trout epithelial cell line RTL-W1 (waterborne, 1 d), activate AhR [71]	1) EC_50_: 6.9 mg/L 2) EC_50_: 46.3–70.5 mg/L 3) EC_50_: 266.7–735.7 mg/L
2-ABTH	1) Zebrafish larvae (semi-static renewal, 144 h), no effect [54] 2) Zebrafish larvae (semi-static renewal, 120 d), neurobehavioral abnormality [55]	1) NEC: 10 μM 2) OEC: 0.05–0.5 mg/L	Zebrafish larvae (static renewal, 120 h), cardiac abnormality [59]	OEC: 0.05–5 mg/L	1) *Xenopus laevis* tadpole thyroid gland, thyroxine (T4) release inhibition [63] 2) Porcine thyroid gland, TPO inhibition [63]	1) IC_50_: 100–300 μM 2) IC_50_: 1200 μM			*Salmonella typhimurium* TA1535/pSK1002 +S9, mortality [73]	LC_50_: 29 mg/L	1) Rainbow trout epithelial cell line RTgill-W1 (waterborne, 12 d), activate AhR [71] 2) Rainbow trout epithelial cell line RTL-W1 (waterborne, 1 d), activate AhR [71]	1) EC_50_: 4.5–86.7 mg/L 2) EC_50_: 463.2–692.7 mg/L
2-SCN-Me-S-BTH	Chinook salmon larvae (flow-through, 48 h), predation avoidance behavior weakened [56]	OEC: 10 μg/L	1) Water flea (static renewal, 7 d), growth inhibition [70] 2) Nematode (24 h), damage reproductive cells [61]	1) NOEC: 2.5 μg/L; EC_50_: 9.64 μg/L 2) OEC: 10 μM					1) Water flea (static nonrenewal, 48 h), mortality [70] 2) Chinook salmon larvae (flow-through, 96 h), mortality [57]	1) LC_50_: 15.3 μg/L 2) LC_50_: 9 μg/L		
2-Me-S-BTH			1) *Phaeodactylum tricornutum* (waterborne, 34 d), growth inhibition [69] 2) Water flea (static renewal, 7 d), growth inhibition [70]	1) LOEC: 10 mg/L; EC_50_: 75 mg/L 2) NOEC: 1.21 mg/L; EC_50_: 6.36 mg/L	Recombinant yeast, activate ER [76]	EC_50_: 7.768 mg/L			1) *Salmonella typhimurium* TA1535/pSK1002 -S9, mortality [73] 2) Water flea (static nonrenewal, 48 h), mortality [70]	1) LC_50_: 48 mg/L 2) LC_50_: 12.7 mg/L		
2-Me-BTH					Recombinant yeast, activate ER [67]	EC_50_: 8 mg/L			*Salmonella typhimurium* TA1535/pSK1002 -S9, mortality [73]	LC_50_: 59 mg/L	Recombinant yeast, activate AhR [67]	EC_50_: 11 mg/L

a The content in the cell of the column where the “End point” was located was given in the form of “species (exposure route, exposure duration), toxic effect”. If the references did not provide one or more of the “exposure route” and “exposure duration”, the missing content was not displayed. OEC, observed effect concentration; LOEC, lowest observed effect concentration; NOEC, no observed effect concentration; NEC, no effect concentration; LC_50_, median lethal concentration; LD_50_, median lethal dose; EC_50_, median effective concentration; ED_50_, median effective dose; IC_50_, median inhibitory concentration.

### 4.1 Neurotoxicity

Epidemiological studies have provided evidence of the neurotoxicity of BTs. A longitudinal cohort study revealed a negative correlation between maternal urinary BT concentrations and offspring psychomotor development index, with this association being more pronounced in male infants. These findings suggested that prenatal exposure to BTs may adversely affect neurodevelopment in children [22]. Another prospective cohort study of 158 pregnant women in Wuhan, China, identified positive correlations between maternal urinary BTs levels and oxidative stress biomarkers (e.g., homocysteine), alongside negative correlations with neuronal function metabolites (e.g., tryptophan), suggesting that BTs may induce neurotoxicity by interfering with the normal function of neurons [77].

The earliest reported evidence of BTsʼ neurotoxicity in model organisms dates back to 1969. Using mice as model organisms, Guess and OʼLeary demonstrated that MBT induced marked neurotoxic responses, including peripheral vasodilation, profuse salivation, and convulsions [53]. Acute high-dose exposure (10 μM, 6 days) to BTH and MBT inhibited central nervous system (CNS) development in zebrafish (*Danio rerio*) larvae and triggered abnormal gene expression patterns, whereas 2-ABTH elicited no comparable effects [54]. However, chronic exposure (120 days) to environmentally relevant doses (50 μg/L) of 2-ABTH was shown to induce oxidative stress in brain tissue, disrupt the synthesis of GABA and 5-HT, and significantly impair zebrafish locomotor, social, anxiety-like, and cognitive behaviors [55], demonstrating its persistent neurological risks. Under simulated flow conditions, Kruzynski et al. exposed chinook salmon (*Oncorhynchus tshawytscha*) to 2-SCN-Me-S-BTH (10 μg/L), followed by a 5-day cohabitation with predators (*Sebastes flavidus*) in estuarine conditions. The exposed salmon exhibited impaired anti-predator behavioral responses, resulting in 5.5-fold higher predation mortality compared to controls [56]. These findings demonstrated that BTsʼ neurotoxicity may have population-level ecological consequences, suggesting potential ecosystem risks. The neurobehavioral effects of 2-SCN-Me-S-BTH on aquatic organisms may stem from gill tissue damage (impairing oxygen transfer) [57] or elevated blood lactate levels [78], thereby suppressing their swimming capacity, underscoring the need for further mechanistic validation.

Beyond the CNS, the sensory nervous system represents another potential target of BTs. Studies have demonstrated that environmentally relevant concentrations (0.1, 0.3, and 0.6 mg/L) of MBT can lead to decreased eye size (eye area/body length) in zebrafish larvae, with histopathological alterations including reduced cellular density in the inner nuclear layer and outer nuclear layer of the retina, as well as decreased lens diameter. These morphological changes subsequently inhibited phototransduction functionality [58]. These results suggested that BTs may induce potential visual toxicity. Given the critical role of vision in essential behaviors such as predation and predator avoidance, BTs-induced visual impairment could significantly impact individual survival. Although current research in this area remains limited, it represents an important avenue for future investigation.

### 4.2 Developmental and reproductive toxicity

Epidemiological studies have found that exposure to BTs is associated with abnormal fetal development. A longitudinal cohort study of 856 mother-child pairs revealed that maternal urinary 2-ABTH levels were positively correlated with femur length and birth length z-scores in female infants, whereas BTH was associated with reduced femur length and birth length z-scores in males. These findings suggested a link between prenatal exposure to BTs and fetal growth parameters, potentially mediated through sex-specific mechanisms [79]. Furthermore, third-trimester maternal urinary 2-ABTH concentrations showed a significant negative correlation with cord blood mitochondrial DNA copy number (mtDNAcn), whereas 2-Me-S-BTH levels were positively associated with mtDNAcn in male infants. These findings indicated that BTs may disrupt fetal energy metabolism and development through mitochondrial interference, with distinct compound-specific mechanisms [80].

In recent years, numerous studies have employed various model organisms to investigate the developmental toxicity of BTs. In zebrafish, larval exposure to 2-ABTH led to cardiac developmental abnormalities in adulthood, including ventricular enlargement, decreased heart rate, and reduced blood flow, accompanied by dysregulation of genes associated with cardiac development and oxidative stress [59]. 2-OH-BTH exposure severely impaired zebrafish larval development, significantly increasing 96-h mortality (LC_50_ = 358 μM) and exacerbating malformations, including notochord deformity, pericardial edema, and yolk sac edema (EC_50_ = 287 μM). Metabolomic analyses further revealed that 2-OH-BTH inhibits the phenylalanine hydroxylation reaction, leading to the accumulation of toxic phenylpyruvate and acetylphenylalanine in zebrafish, which may serve as a key trigger for developmental abnormalities [25]. Similar toxic effects were also observed in fathead minnows (*Pimephales promelas*) exposed to TWP leachate, including decreased heart rate, hatching success, and body length in larvae, as well as severe malformations. These effects may be associated with several chemicals, including BTH, 2-ABTH, and MBT [81]. In bivalves (*Mytilus galloprovincialis*), multiple endpoints were affected to varying degrees after exposure to tire rubber leachate (BTH: 1460–2313 μg/L), including embryonic development, larval motility, and larval survival [3]. In avian species, injection of crumb rubber leachate into the yolks of chicken embryos resulted in developmental abnormalities after 7 days of incubation, including malformations, growth retardation, and impaired brain and cardiovascular systems. Similar abnormalities were observed when embryos were exposed to 1.5 mg/L BTH (equivalent to the leachate concentration) alone, suggesting that BTH may be a key contributing component to the observed leachate toxicity [82]. Similar developmental abnormalities were observed in chicken embryos injected with MBT into the air sac, including developmental defects in the eye, wing, spinal cord, and other parts [60].

Reproductive toxicity of BTs has also been observed across multiple species. For Catalan chub (*Squalius laietanus*) inhabiting waters downstream of an industrial wastewater treatment plant, both male and female fish exhibited a significantly lower gonadosomatic index compared to upstream populations, accompanied by asynchronous ovulation and reduced vitellogenic oocytes in females, along with decreased seminiferous tubule diameter in males. These abnormalities may be associated with the elevated downstream BTH concentrations (downstream: 61–506 ng/L vs upstream: <3 to 50 ng/L) [26]. Exposure to 10 μM 2-SCN-Me-S-BTH activated the p53/CEP-1 signaling pathway, inducing germ cell apoptosis in *Caenorhabditis elegans*. This led to chromosomal abnormalities in oocytes during diakinesis of meiotic prophase I and impaired chromosome segregation in early embryos [61].

However, several mammalian model studies did not show significant developmental and reproductive toxicity of BTs. Daily intraperitoneal injection of MBT (200 mg/kg) to pregnant rats from gestation day 1 to 15 did not induce adverse effects on offspring survival, body length, or body weight, nor did it cause teratogenic effects [62]. Ema et al. reported that maternal exposure to dibenzothiazyl disulfide (MBTS) at the maximum tested dose [596 mg/(kg⋅d)] via oral gavage produced no adverse developmental effects in offspring [83]. These findings demonstrate species-specific differences in BT toxicity, which may be further modulated by exposure route, exposure dose, and other factors. This highlights the necessity for holistic toxicity assessment strategies, particularly for human health risk assessment, where single-species testing may inadequately reflect actual toxicological profiles.

### 4.3 Endocrine disruption

Endocrine disruption is also one of the common toxic effects of BTs. Epidemiological investigations have established significant associations between BTs exposure and endocrine-metabolic disorders in human populations, with particularly pronounced effects observed in sensitive subgroups such as pregnant women. In a cohort of 459 pregnant women, four BTs (BTH, 2-OH-BTH, 2-Me-S-BTH, and 2-ABTH) were significantly associated with disruptions in sex hormone levels (17β-estradiol, estriol, testosterone, etc.) during pregnancy, and these associations were more pronounced in women carrying male fetuses [23]. Another prospective cohort study of 1770 pregnant women revealed that elevated urinary 2-OH-BTH levels during early pregnancy were significantly associated with disrupted glucose homeostasis and increased risk of gestational diabetes mellitus, suggesting certain BTs may act as potential insulin-regulating disruptors [84].

Both *in vivo* and *in vitro* studies have demonstrated that BTs exhibit multiple characteristic endocrine-disrupting modes, including interference with hormone biosynthesis and transport, as well as modulation of hormone receptor activity. Using *Xenopus laevis* tadpoles as a model organism, Hornung et al. [63] demonstrated that even low-dose MBT exposure (18 μg/L) significantly reduced both triiodothyronine (T3) and thyroxine (T4) levels in thyroid tissue. Through integrated *ex vivo* thyroid explant and microsomal assays, the team further identified multiple BTs, including 2-ABTH, BTH, and 5-chloro-2-mercaptobenzothiazole (CMBT), as thyroid peroxidase (TPO) inhibitors that disrupt the synthesis of T3/T4. Their inhibitory potency followed the order: CMBT > MBT > 2-ABTH > BTH [63,64]. In toxicity studies using fathead minnow and zebrafish models, MBT exposure similarly suppressed TPO transcriptional expression and reduced T3/T4 levels, indicating conserved thyroid disruption mechanisms through TPO inhibition across fish species [65,66]. Using recombinant yeast assays coupled with luciferase reporter systems, researchers further identified BTH and 2-Me-BTH as potent agonists of multiple nuclear hormone receptors, including estrogen receptors (ER) [67] and glucocorticoid receptors [85].

### 4.4 Immunotoxicity

Several BTH derivatives have shown potent anti-inflammatory activity and have been reported to mitigate excessive immune responses. These pharmacological effects predominantly depend on strong electron-withdrawing groups and lipophobic substituents (e.g., triazoles, aryl groups, etc.) at the 2-position of the thiazole ring and 4/5/6-positions of the benzene moiety [86]. This review primarily focuses on the parent BTH scaffold and its derivatives bearing only weakly polar substituents. For these compounds, anti-inflammatory activity was not observed; instead, they exhibited measurable immunotoxicity. In a clinical investigation of tennis shoe-induced contact dermatitis, patch testing coupled with chromatographic separation identified MBT and its dimer (MBTS) as the causative allergens [24]. Furthermore, investigators directly assessed the sensitization potential of MBT through controlled animal exposure studies. Using the murine local lymph node assay, Ikarashi et al. evaluated the sensitization potential of four rubber additives, including MBT, and demonstrated that MBT induced moderate lymphocyte proliferation responses [68]. Sensitization reactions were also confirmed in guinea pig models, where both 5% and 10% MBT concentrations elicited dermal reactivity [87]. Mechanistic studies on MBT sensitization have identified the thiol (-SH) group as the critical functional moiety, which contributes predominantly to allergenic potential through interactions with amino acid residues [88,89]. Moreover, a recent study has reported that 2-SCN-Me-S-BTH in leather products can also cause contact dermatitis in humans [90].

### 4.5 Potential impacts on ecosystems

Since BTs distribute into soil compartments, organisms in the soil ecosystem may potentially get exposed to BTs. In a 21-day soil TWPs exposure experiment, woodlice (*Porcellio scaber*) and enchytraeid worms (*Enchytraeus crypticus*) showed minor effects, with only slight inhibition (18%–20% reduction) observed in enchytraeid wormsʼ reproduction. In contrast, springtails (*Folsomia candida*) were more significantly affected, exhibiting 24% and 38% reductions in survival and reproduction rates, respectively, at 1.5% (w/w) TWPs. As the predominant organic compound in TWPs, BTH (0.0195 mg/kg) contributed substantially to the observed toxicity [91]. Furthermore, exposure to 1000 mg/kg TWPs significantly reduced soil fungal biomass and altered community structure, and these effects were replicated by 200 ng/kg BTH and 2-OH-BTH (the dominant BTs in soil), suggesting that both compounds may contribute to these adverse impacts [92].

The aforementioned studies have demonstrated that BTs have multiple toxic effects on fish. Phytoplankton and zooplankton, as the basic components of the aquatic food web, are equally crucial for maintaining the balance of the ecosystem. Growing evidence has confirmed that BTs also show significant impacts on these planktonic organisms. BTH, 2-OH-BTH, and 2-Me-S-BTH inhibited growth in three algal species (*Dunaliella tertiolecta*, *Raphidocelis subcapitata*, and *Phaeodactylum tricornutum*), with EC_50_ ranges of 41 to >100 mg/L, 16 to >100 mg/L, and 75 to >100 mg/L, respectively. Among them, 2-OH-BTH exhibited the strongest inhibitory effects [69]. Certain benzothiazolium salts have been shown to disrupt chlorophyll biosynthesis in *Euglena gracilis*, inducing permanent chloroplast loss and triggering albino mutations [93,94]. Acute exposure (48 h) to TWP leachate containing BTH as the primary organic component (24–91 μg/L) resulted in 100% mortality in *Daphnia magna* [2]. Comparative toxicity assessment revealed differential effects of five BTs (2-SCN-Me-S-BTH, MBT, 2-Me-S-BTH, BTH, and 2-OH-BTH) on *Ceriodaphnia dubia*, with 2-SCN-Me-S-BTH being the strongest (acute EC_50_ = 15.3 μg/L; chronic EC_50_ = 9.64 μg/L) [70]. In addition, the toxic effects of BTs have been observed in a greater variety of aquatic plants [95,96] and animals [97,98]. However, toxicity assessments of TWP leachates using *D. magna*, *Lemna minor*, and *Chlorella vulgaris* revealed acute effects only in *D. magna*, with minimal toxicity observed in the *L. minor* and *C. vulgaris* [99]. Yang et al. further demonstrated that co-existing BTH in TWPs leachates attenuated zinc (Zn) toxicity in *Tigriopus japonicus* [100].

### 4.6 Potential toxic targets of BTs

Identifying toxic targets of pollutants is fundamental for constructing adverse outcome pathways (AOPs) and conducting risk assessments. Although numerous toxic effects of BTs have been documented, research on their mechanisms of toxicity and key toxic targets remains limited. Therefore, this section integrates three major target prediction databases (TargetNet, SwissTargetPrediction, and ChEMBL), the Tox21 toxicity database, and the available literature to discuss the potential toxic targets of BTs.

**Fig. 4:**
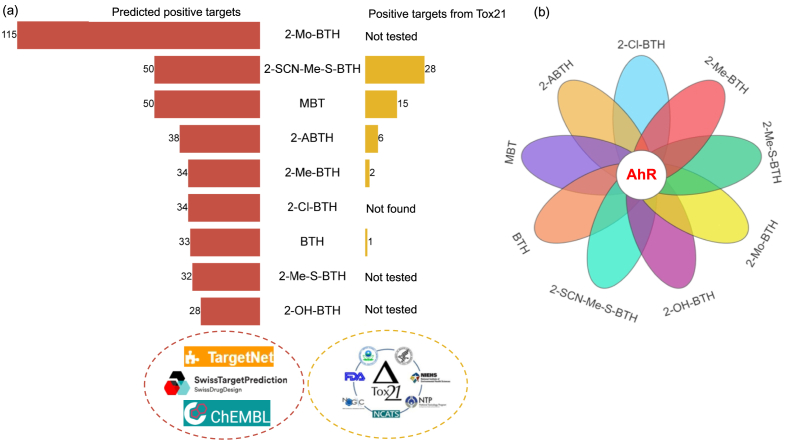
(a) The overall screening results of the potential targets of BTs. The left panel reveals the predicted targets of BTs using TargetNet, SwissTargetPrediction, and ChEMBL. The right panel shows the experimentally validated targets of BTs from the Tox21 database. “Not found” means the chemicals were tested, but no positive targets were reported. “Not tested” means the chemicals were not examined. (b) The Venn diagram of the shared targets of selected BTs.

Fig. 4 illustrates the screening process and results for exploring shared toxic targets of BTs, with the specific target categories from three target prediction databases and the Tox21 toxicity database detailed in Table S3. Firstly, potential toxic targets of individual BTs were identified using three major target prediction databases. Subsequently, three shared toxic targets among BTs were identified through screening, including AhR (Aryl Hydrocarbon Receptor), RELA (RELA Proto-Oncogene, NF-κB Subunit), and RORA (RAR-Related Orphan Receptor A). Finally, a comparative analysis of the Tox21 toxicity database identified the AhR as a potential shared toxic target of BTs. AhR is a ligand-dependent transcription factor that regulates xenobiotic metabolism and various physiological processes. A variety of pollutants, like 2,3,7,8-Tetrachlorodibenzo-p-dioxin (TCDD), can bind to AhR and trigger the nuclear translocation of AhR, which then forms a heterodimer with aryl hydrocarbon receptor nuclear translocator (ARNT), which subsequently initiates the transcription of downstream genes such as phase I metabolic enzymes (cytochrome P450 1A: CYP1A) and phase II metabolic enzymes (glutathione S-transferases). These molecular changes further lead to oxidative stress, apoptosis, and mitochondrial dysfunction, and ultimately result in adverse outcomes like developmental abnormalities [101], embryonic lethality [102], and reproductive impairment [103].

Multiple *in vitro* and *in vivo* toxicity studies have also corroborated that BTs can activate AhR. Zeng et al. demonstrated that BTs induced both cytotoxicity and oxidative stress in two rainbow trout epithelial cell lines (RTgill-W1 and RTL-W1). In RTL-W1 cells, CYP4501A was significantly induced by 2-ABTH and 2-OH-BTH, indicating BTsʼ capacity to alter xenobiotic metabolism and activate AhR [71]. In another investigation of tire extract toxicity, researchers evaluated AhR agonist activity in four commercial BTs (BTH, 2-Me-S-BTH, MBT, and 2-OH-BTH) using recombinant mouse hepatoma cells and gel retardation assays. The results demonstrated that all tested BTs stimulated AhR transformation and DNA-binding activity, but only MBT and 2-OH-BTH induced AhR-dependent gene expression in recombinant mouse hepatoma cells [104]. Recombinant yeast assays further confirmed the AhR-binding and AhR-activating potential of BTH, 2-Me-S-BTH, and 2-OH-BTH, with EC_50_ values of 10.2, 11.0, and 6.9 mg/L, respectively [67]. In addition to *in vitro* cell experiments, similar findings have also been reported *in vivo*. Oral administration of BTH [1 mmol/(kg⋅d) for 5 consecutive days] significantly induced CYP4501A1 and CYP4501A2 enzyme activities in rats, as confirmed by spectral analysis, demonstrating potential systemic AhR pathway activation [105]. Furthermore, Zhang et al. employed molecular dynamics simulations to demonstrate that MBT induces conformational changes in the AhR ligand-binding domain. The quantitative real-time polymerase chain reaction (qPCR) and Western Blot assay confirmed AhR transcriptional activation by MBT, resulting in about 1.5-fold upregulation of both CYP1A1 and CYP1B1 [106].

Collectively, these *in vitro* and *in vivo* studies demonstrate that BTs may induce toxicity by activating the AhR pathway. However, the precise mechanistic details remain uncharacterized, warranting further investigation into their toxicological and biological implications.

## 5 Environmental transformation products of BTs and their predicted toxicity

### 5.1 Environmental transformation products of BTs

The environmental transformation pathways of BTs mainly include chemical transformation, biological transformation, and photolysis, and their corresponding transformation products are summarized in Table 2 [[107], [108], [109], [110], [111]]. For chemical transformation, BTs generally undergo hydroxylation and removal of other functional groups during ozonation [112], persulfate oxidation [107], and UV irradiation [108], whereas chlorination results in chlorinated transformation products [109]. Biological treatment of BTs also mainly leads to hydroxylated transformation products [113]. It should be noted that natural photolysis not only induces hydroxylation of BTs but also triggers methylation reactions, leading to the formation of toxic BTs (e.g., MBT) [110].

**Table 2:** Summary of the predicted toxicity of different transformation products^a^.

Parent compound	Structural formula	Predicted acute toxicity	Predicted chronic toxicity	Treatment process	Transformation products	Structural formula	Predicted acute toxicity^b^ (mg/L)			Predicted chronic toxicity (mg/L)		
							Fish	Daphnid	Green algae	Fish	Daphnid	Green algae
BTH		Fish 96 h LC_50_: 78.3 mg/L Daphnid 48 h LC_50_: 45.2 mg/L Green algae 96 h EC_50_: 35.9 mg/L	Fish: 7.8 mg/L Daphnid: 4.6 mg/L Green algae: 9.73 mg/L	UV/chlorine [111]	TP1		236	130	86.3	22.3	11.7	21.2
					TP2 (2-OH-BTH)		59.8	35.1	29.9	6.07	3.75	8.42
					TP3 (2-Cl-BTH)		25.9	15.9	16.1	2.77	1.91	4.99
					TP4		706	372	205	63.3	29.6	45.6
					TP5		873	458	247	77.7	35.9	54.2
					TP6		19.4	12.1	13.1	2.11	1.52	4.23
					TP7		172,000	13.549	184.558	1099.879	3.354	226.189
					TP8		446,000	178,000	30,902.09	28,771.2	6513.27	3692.72
				UV/persulfate [107]	TP9		706	372	205	63.3	29.6	45.6
					TP10		873	458	247	77.7	35.9	54.2
2-SCN-Me-S-BTH		Fish 96 h LC_50_: 19.4 mg/L Daphnid 48 h LC_50_: 12.2 mg/L Green algae 96 h EC_50_: 13.9 mg/L	Fish: 2.14 mg/L Daphnid: 1.59 mg/L Green algae: 4.59 mg/L	Natural sunlight radiation [110]	TP11 (MBT)		23.1	14.2	14.7	2.48	1.73	4.6
					TP12 (BTH)		78.3	45.2	35.9	7.8	4.6	9.73
					TP13 (2-OH-BTH)		59.8	35.1	29.9	6.07	3.75	8.42
					TP14		31.6	19.1	18.7	3.33	2.24	5.65
					TP15		180	101	71.3	17.3	9.5	18.1
					TP16		1.12	0.812	1.67	0.146	0.157	0.755
					TP17		266	148	100	25.4	13.6	25.1
2-ABTH		Fish 96 h LC_50_: 123 mg/L Daphnid 48 h LC_50_: 69.8 mg/L Green algae 96 h EC_50_: 52 mg/L	Fish: 12 mg/L Daphnid: 6.81 mg/L Green algae: 13.6 mg/L	Chlorine [109]	TP18		39.8	24	22.9	4.18	2.76	6.85
					TP19		12.5	7.98	9.73	1.4	1.08	3.32
					TP20		1090	564	292	95.6	43	62.7
					TP21		117	67.4	53.5	11.6	6.86	14.5
MBT		Fish 96 h LC_50_: 23.1 mg/L Daphnid 48 h LC_50_: 14.2 mg/L Green algae 96 h EC_50_: 14.7 mg/L	Fish: 2.48 mg/L Daphnid: 1.73 mg/L Green algae: 4.6 mg/L	UV [108]	TP22		855,000	368,000	87,700	60,300	16,700	12,400
					TP23		236	130	86.3	22.3	11.7	21.2
					TP24		236	130	86.3	22.3	11.7	21.2
					TP25		236	130	86.3	22.3	11.7	21.2
					TP26 (2-OH-BTH)		59.8	35.1	29.9	6.07	3.75	8.42
					TP27		1210	613	288	103	43.8	58.9
					TP28 (BTH)		78.3	45.2	35.9	7.8	4.6	9.73
					TP29		150	84.6	62.1	14.6	8.17	16.1
					TP30		c					
					TP31							
					TP32							
					TP33							
					TP34		13,800	6390	2070	1060	356	346
					TP35							
					TP36							
					TP37							
					TP38							
					TP39							
					TP40							
					TP41							
					TP42							
					TP43							

a The acute and chronic toxicity values for both parent compounds and transformation products were predicted using the ECOSAR v2.2 model.

b The acute toxicity indicators of different species were fish (96 h LC_50_), daphnid (48 h LC_50_), and green algae (96 h EC_50_), respectively.

c Blank cells in the table indicate that the specific chemical structures of the compounds were not provided in the references, rendering predictive software tools inapplicable.

**Fig. 5:**
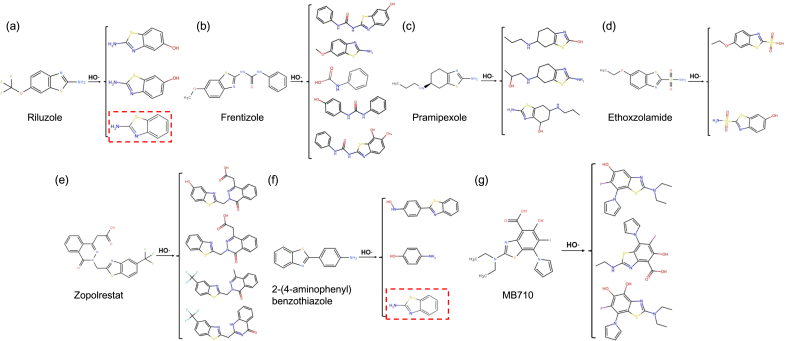
Prediction of transformation products for seven BT-derived pharmaceuticals. (a) Riluzole, (b) Frentizole, (c) Pramipexole, (d) Ethoxzolamide, (e) Zopolrestat, (f) 2-(4-aminophenyl)benzothiazole, and (g) MB710. Reaction conditions: reacted with HO**·**, no energy input, and the influence of pH value not considered.

Owing to their favorable bioactivity, BTs serve as precursors for various pharmaceuticals; however, the environmental transformation of such BT-derived pharmaceuticals has not yet been investigated. We randomly selected seven commercial BT-derived pharmaceuticals to explore their potential transformation products with an open-access computational prediction model (Fig. 5). Among these, transformation products of two pharmaceuticals (Riluzole and 2-(4-aminophenyl)benzothiazole) were found to contain 2-ABTH (highlighted with red dashed boxes), suggesting that pharmaceutical transformation may serve as a potential source of toxic BTs in the environment. Therefore, future studies should place greater emphasis on investigating the environmental fate of these pharmaceuticals and conducting experimental validation to further verify the predicted results.

In summary, BTs can generate a variety of transformation products through diverse transformation pathways. Among these, BTH and 2-OH-BTH demonstrate high detection frequencies, representing the predominant transformation products formed during the environmental transformation of BTs. Moreover, 2-OH-BTH is identified as a thermal oxidation product of BTH during the incineration of rubber materials [38]. Consequently, future studies on BTsʼ transformation products should prioritize the investigation of BTH and 2-OH-BTH due to their environmental persistence. It is also worth noting that the model prediction results show that BT-derived pharmaceuticals may transform into BTs, thus becoming a potential source of BTs in the environment.

### 5.2 Predicted toxicity of transformation products of BTs

It has been reported that some transformation products of environmental pollutants retain the active structures of their parent compounds, thereby exhibiting similar toxicity [114]. As shown in Table 2, BTs generate numerous transformation products during environmental transformation, yet the toxicity of these byproducts is still largely unstudied. Recent studies have applied a prediction model to evaluate the toxicity of several transformation products. Here, we employed the same prediction model (ECOSAR v2.2) to supplement and standardize toxicity prediction for all documented transformation products. As anticipated, the predicted toxicity of most transformation products was lower than or comparable to that of their parent compounds [107,110,[115], [116], [117], [118], [119]]. However, certain transformation products were predicted to demonstrate significantly enhanced toxicity compared to the original BTs. Among the eight transformation products (TP; TP1–TP8) generated during UV/chlorine treatment of BTH, three transformation products (TP2, TP3, and TP6) exhibited higher predicted toxicity than the parent compound [111]. Furthermore, transformation products with varying toxicity levels were detected in chlorinated 2-ABTH solutions, displaying the following toxicity order: chlorinated derivatives > parent compound > hydroxylated derivatives [109]. The predicted toxicity of transformation products correlates strongly with molecular structure. Hydroxylation on the benzene ring generally reduces toxicity (e.g., BTH > TP1; 2-ABTH > TP20; 2-OH-BTH > TP29), with progressive attenuation observed upon additional hydroxylations (TP5 < TP4 < TP1). Conversely, thiazole ring hydroxylation increases toxicity, as evidenced by the greater toxicity of 2-OH-BTH relative to BTH. Chlorination consistently enhances transformation productsʼ toxicity regardless of reaction site, whether on the benzene ring (TP18 > 2-ABTH) or thiazole ring (2-Cl-BTH > 2-ABTH). Toxicity shows positive correlation with chlorination degree (TP19 > TP18), suggesting additive effects of multiple Cl-substituents. The introduction of chlorine atoms and hydroxyl groups to the benzene and thiazole rings, respectively, generates transformation products with significantly enhanced toxicity (e.g., TP6 > 2-OH-BTH/2-Cl-BTH). However, the hydroxylation of chlorinated derivatives on the benzene ring effectively mitigates toxicity, reducing it to levels comparable to the parent compounds (e.g., TP18 > TP21 ≈ 2-ABTH). Ring-opening reactions significantly reduce the toxicity of the parent compounds, as exemplified by thiazole ring cleavage products (TP7, TP8, and TP27). While chlorination effectively degrades parent compounds, it may concurrently generate more toxic transformation products, particularly chlorinated derivatives. Consequently, comprehensive identification and toxicity assessment of transformation products must precede the selection of chlorination treatments to mitigate potential secondary environmental risks.

Beyond acute and chronic toxicity, emerging studies have investigated additional adverse effects of transformation products. Among 22 transformation products derived from MBT UV photolysis, all exhibited reduced acute/chronic toxicity relative to the parent compound. However, 18 demonstrated higher TPO inhibition probabilities, and three 2-OH-BTH isomers (TP23–TP25) showed enhanced estrogenic activity [108]. These findings reveal a toxicity trade-off: while conventional toxicity metrics may improve, endocrine disruption potential escalates, underscoring the necessity for multiplexed toxicity assessment in transformation products. Notably, hydroxylated BT derivatives (particularly those formed via benzene ring hydroxylation) demonstrated superior environmental stability across multiple transformation systems. These compounds exhibited remarkable resistance to degradation, enabling prolonged environmental persistence, with trihydroxylated products showing the highest resilience [107,108,111,116]. Therefore, future research must prioritize tracking these stable hydroxylated transformation products in transformation systems, coupled with comprehensive toxicological profiling to establish their real-world hazard potential. This dual approach will significantly enhance the reliability and accuracy of risk assessments for BTsʼ transformation products.

## 6 Conclusions and perspectives

Concerns regarding BTs have increased in recent years, and substantial progress has been made in understanding their distribution and toxicity. However, several research gaps remain to be addressed, including the source apportionment of emissions of BTs, the identification of transformation products, the assessment of exposure risks, and the elucidation of toxic mechanisms.

A variety of BTs have been widely detected in various environmental media (ranging from n.d. to tens of thousands of ng/L), among which BTH and 2-OH-BTH are frequently identified at relatively high concentrations, likely due to their extensive use [36], strong leaching capacity [120], and stable molecular structures [121]. Furthermore, BTH and 2-OH-BTH are resistant to current wastewater treatment processes [11,122] and can be generated through the transformation of other BTs [108,110]. Therefore, particular attention needs to be paid to these two BTs in the future. In addition, knowledge regarding the source apportionment and historical accumulation processes of BTs in the environment remains largely unexplored; it is of great urgency to clarify the spatiotemporal distribution trends of BTs in different environmental media. Using computational models, we further found that several BT-derived pharmaceuticals might be transformed into BTs of concern during wastewater treatment, like 2-ABTH, highlighting a potentially neglected source of BTs. Investigating the environmental occurrence and fate of such BT-derived pharmaceuticals is of great importance to provide deeper insight into the source apportionment of BTs.

BTs can undergo diverse reactions and generate numerous transformation products according to the environmental conditions. However, accurate identification of these transformation products remains challenging due to limitations in analytical methods. As two high-throughput analytical approaches, non-target analysis (NTA) and suspect screening analysis (SSA) have been increasingly applied to the large-scale screening of environmental contaminants [[123], [124], [125]]. The combined use of these two approaches (NTA + SSA) can compensate for their respective limitations, and their integration with artificial intelligence (NTA + SSA + AI) is expected to further enhance the efficiency and accuracy of identification, thereby providing a promising solution for the high-throughput screening of transformation products.

BTs have been detected in various human matrices, particularly in urine, serum, adipose tissue, and breast milk. Consistent with their environmental occurrence, BTH and 2-OH-BTH are also the primary compounds detected in human samples. However, it should be noted that, in addition to industrial sources (e.g., tires), BTH exists as a natural compound in food, beverages, and tobacco [[126], [127], [128]], which can also contribute to the high exposure level to BTH. Comparative studies across demographic groups have revealed age-dependent variations in BT exposure, with elders and children often exhibiting higher exposure levels than adults. Consequently, more attention should be paid to assessing the health impacts of BTs on these vulnerable populations in future research. At the same time, it is necessary to establish health-based guidance values that account for population sensitivity to better protect different groups of people. Human exposure to BTs occurs mainly through ingestion, inhalation, and dermal contact. Compared to ingestion and inhalation, current research on dermal exposure remains limited, with most studies focusing primarily on clothing-related pathways. Due to their ultraviolet absorption and antibacterial properties, some BTH derivatives have been reported to be used in personal care products (such as sunscreens) or as preservatives in them [129,130]. However, the actual dermal exposure from the use of personal care products remains unclear, necessitating further investigation.

Epidemiological and toxicological evidence indicate that BTs exhibit a broad spectrum of toxic effects, including neurotoxicity, developmental and reproductive toxicity, endocrine-disrupting effects, and immunotoxicity. Among the reported BTs, MBT showed greater potency in inducing toxic effects. However, current toxicological studies mainly employ fish models to explore the toxic effects, which might lead to uncertainty in health risk assessments. For example, fish model studies showed that MBT could induce significant developmental defects, whereas mammalian model studies did not observe developmental toxicity of MBT [60,64,68]. Therefore, more toxicological studies using organoids or organ-on-a-chip as models should be conducted in the future to promote accurate health risk assessment. Moreover, present studies regarding the risk assessments of BTs were often limited to the parent compounds, while neglecting the transformation products. According to the ECOSAR toxicity prediction, the chlorinated transformation products generated during chlorination possibly exhibit increased toxicity. Therefore, more efforts should be devoted to assessing the exposure levels and the risks posed by these transformation products.

Regarding the mechanisms of toxicity, several studies have indicated that BTs can induce oxidative stress and alter the expression of specific genes, thereby leading to associated toxic effects [59,71,77,131]. Based on toxicology database screening and published findings, AhR was highlighted as a potential shared toxic target of BTs. Various studies have shown that BTs can stimulate the expression of AhR-related genes and CYP450 enzymes; however, the mechanistic understanding of the activation of AhR signaling is still insufficient. The upregulation of CYP450 enzymes is not a necessary and sufficient indicator of AhR pathway activation [132]. Therefore, the translocation of AhR to the nucleus, the hallmark of AhR signaling activation, should be further verified. In addition, the subsequent downstream biological events of BTs-induced AhR signaling activation remain unclear. Following the activation of AhR and CYP450 enzymes, excessive reactive oxygen species (ROS) can be generated during the CYP450-mediated oxidative reactions, further promoting lipid peroxidation and leading to ferroptosis. Additionally, AhR signaling intricately interacts with other critical signaling pathways such as the Nrf2-Gpx4 axis, thereby regulating the progression of ferroptosis [133]. To comprehensively reveal the toxic mechanisms of BTs, multi-omics strategies, such as transcriptomics and lipidomics, should be applied to explore the toxicological events induced by BTs, and the resulting hypotheses should then be verified through gene-editing technologies (e.g., CRISPR-Cas9), ultimately constructing the AOP for BTs.

The extensive environmental presence and potential toxic effects of BTs have drawn the attention of governments, and some countries have taken certain measures to strengthen the regulation of such chemicals. The German Environmental Protection Agency has classified BTH as a potentially persistent, mobile, and toxic (PMT) substance [134]. In China, MBT has been included in the list of “high-pollution and high-environmental risk” products [135]. The New York State Department of Environmental Conservation has set a standard of 50 μg/L for MBT in surface water [136]. Canada is also promoting the inclusion of MBT in its environmental protection law and taking control measures against it [137]. Although some countries have taken actions to regulate BTs, most of them are concentrated on MBT. Given the diverse types and large-scale applications of BTs, it is necessary to investigate the regulatory necessity of more BTs in the future, especially BTH and 2-OH-BTH, which are predominantly distributed in the environment. In addition, more human exposure risk assessments are also urgently needed to provide a basis for the risk management of BTs and the formulation of related policies.

## 7 Limitations

BTs are a group of emerging contaminants, and the relevant studies are still limited. This review provides a relatively comprehensive summary of the environmental occurrence, exposure, toxicity, and transformation of BTs via the combination of a literature survey and computational models. Based on the prediction results of computational models, we highlighted that BT-derived pharmaceuticals could be transformed into the BTs of concern, and the halogenated transformation products possibly exhibited higher toxicity. However, experimental validation is still not available for the proposed speculation. There is an urgent need for solid experimental data regarding the transformation process of BTs and the toxicity of their transformation products. In addition, we followed a systematic literature search and screening process to comprehensively cover the literature related to the topic of this review, yet the literature search was limited to the Web of Science database and the English language, which might have omitted studies in other databases or languages. These limitations mean that this review may not cover all relevant studies under this topic, thereby introducing potential biases. Future review studies can consider expanding the search scope to more databases and languages, and updating rigorous experimental studies to promote our understanding of this research field.

## CRediT authorship contribution statement

**Junʼan Bao:** Writing – original draft, Validation, Methodology. **Sheng Wei:** Writing – review & editing, Supervision, Funding acquisition. **Ting Xu:** Validation, Funding acquisition. **Miao Cao:** Methodology, Investigation. **Huan Wang:** Validation, Data curation. **Yiqun Song:** Methodology, Data curation. **Taoqin Chen:** Investigation. **Daqiang Yin:** Writing – review & editing, Supervision, Project administration, Funding acquisition, Conceptualization.

## Declaration of competing interest

The authors declare that they have no known competing financial interests or personal relationships that could have appeared to influence the work reported in this paper.
