# Supplementary material for: Environmental occurrence, exposure, toxicity, and transformation of benzothiazoles: A review
Source: Eco Environ Health. 2026 Apr 24;5(2):100243. doi: 10.1016/j.eehl.2026.100243 (PMC13213317; doi:10.1016/j.eehl.2026.100243)
Supplement: Multimedia component 1 [file mmc1.docx]

Supplementary Information for

**Environmental occurrence, exposure, toxicity and transformation of benzothiazoles: A Review**

Jun'an Bao ^a^, Sheng Wei ^a, b, *^, Ting Xu ^a, c^, Miao Cao ^a, b^, Huan Wang ^a^, Yiqun Song ^a^, Taoqin Chen ^d^, Daqiang Yin ^a, c, *^

^a^ Key Laboratory of Yangtze River Water Environment, Ministry of Education, College of Environmental Science and Engineering, Tongji University, Shanghai 200092, China

^b^ Postdoctoral Research Station of Environmental Science and Engineering, Tongji University, Shanghai 200092, China

^c^ Shanghai Institute of Pollution Control and Ecological Security, Shanghai 200092, China

^d^ Shanghai Center for Energy-saving and Emission Reduction, Shanghai 200232, China

* Corresponding authors.

E-mail: weisheng@tongji.edu.cn (S. Wei), yindq@tongji.edu.cn (D. Yin)

This file includes:

**Table S1. Occurrence of BTs in different media.**

**Table S2. Occurrence of BTs in different human matrices.**

**Table S3. Specific types of BTs positive targets.**

**References**

Table S1. Occurrence of BTs in different media ^a^.

| **Matrix** | **Region** | **Date** | **N** | **Unit** | **BTH** | **MBT** | **2-OH-BTH** | **2-ABTH** | **2-Me-S-BTH** | **2-Me-BTH** | **2-SCN-Me-S-BTH** | **2-Cl-BTH** | **2-Mo-BTH** | **Total** | **Ref** |
| --- | --- | --- | --- | --- | --- | --- | --- | --- | --- | --- | --- | --- | --- | --- | --- |
| Surface water | Guangzhou, China | 2020 | 19 | ng/L | 39.6~119 (73.9, 100%) |  |  | n.d.~3.65 (0.8, 78.9%) | n.d.~13.7 (3.64, 52.6%) | n.d.~13.8 (0.73, 5.3%) |  |  |  |  | [1] |
| Surface water | Pearl River estuary, China | 2021 | 8 | ng/L | 60~628 (100%) |  | n.d.~31 (87.5%) |  | 9.4~54 (100%) |  |  |  |  |  | [2] |
| Surface water | North River, Pearl River basin, China | 2018 | Dry season: 13 Wet season: 13 | ng/L | Dry season: n.d. Wet season: n.d.~14.5 (38%) | Dry season: 16.6~109 (100%) Wet season: n.d.~413 (62%) | Dry season: 11.3~249 (100%) Wet season: 17.1~143 (100%) |  | Dry season: 17.3~19.7 (100%) Wet season: n.d.~6.94 (54%) | Dry season: n.d. Wet season: 0.32~0.4 (100%) |  |  |  |  | [3] |
| Surface water | West River, Pearl River basin, China | 2018 | Dry season: 22 Wet season: 22 | ng/L | Dry season: n.d.~63.6 (64%) Wet season: n.d.~123 (55%) | Dry season: 16.3~200 (100%) Wet season: 10.8~1112 (100%) | Dry season: 17.3~426 (100%) Wet season: 39.1~246 (100%) |  | Dry season: n.d.~20.7 (86%) Wet season: n.d.~48.2 (73%) | Dry season: n.d.~59.5 (64%) Wet season: n.d.~135 (55%) |  |  |  |  | [3] |
| Surface water | Pearl River, China | 2017~2018 | Dry season: 30 Wet season: 30 | ng/L | Dry season: 213~1082 (545) Wet season: 30~574 (268) |  | Dry season: 12~128 (55) Wet season: 10~75 (19) |  | Dry season: 35~314 (138) Wet season: 30~270 (100) |  |  |  | Dry season: 36~204 (109) Wet season: 42~252 (97) |  | [4] |
| Surface water | Pearl River estuary, China | 2022 | Dry season: 18 Wet season: 18 | ng/L | Dry season: 19.4~99.6 (100%) Wet season: 42.9~285 (100%) |  | Dry season: 23.1~191 (100%) Wet season: 34.7~1652 (100%) | Dry season: ＜0.09~0.29 (72.2%) Wet season: 0.14~2.43 (94.4%) | Dry season: 5.57~41.8 (100%) Wet season: 8.75~39.8 (100%) |  |  |  |  |  | [5] |
| Surface water | Pearl River, China | 2015 | 13 | ng/L | 68.8~502 (241, 100%) | 6.12~422 (75.2, 100%) | 111~1993 (362, 100%) | 2.3~22.4 (8.73, 100%) | 92.3~738 (348, 100%) |  |  |  |  |  | [6] |
| Surface water | Dongjiang River, China | 2015 | 13 | ng/L | 36.5~319 (139, 100%) | 1.67~196 (26.7, 64%) | 81.4~971 (250, 100%) | 0.84~167 (13.38, 100%) | 12.8~438 (145, 100%) |  |  |  |  |  | [6] |
| Surface water | Queensland, Australia | 2021 | 21 | ng/L |  |  | ＜3~217 (62%) | 0.08~30 (100%) | ＜3~172 (86%) |  |  |  | ＜0.1~5.7 (90%) |  | [7] |
| Surface water | Schwarzbach watershed, Germany | 2008~2010 | 15 | ng/L | 58~856 (100%) |  |  |  |  |  |  |  |  |  | [8] |
| Surface water | Catalonia, Spain | — | 4 | ng/L | n.d.~60 (75%) |  | 6~45 (100%) | 4~25 (100%) |  |  |  |  |  |  | [9] |
| Surface water | Queensland, Australia | 2020 | 32 | ng/L |  |  | 2.2~449 (100%) | 4.6~77 (100%) | 4.5~274 (100%) |  |  |  | ＜0.7~6.6 (84.4%) |  | [10] |
| Surface water | Kerala, India | 2008, 2011 | 7 | ng/L | 60~500 (100%) |  | n.d.~900 (14.3%) |  | 10~7400 (100%) | n.d.~100 (71.4%) |  |  |  |  | [11] |
| Surface water (river) | Taoyuan, Taiwan, China | — | 3 | ng/L | n.d.~3100 (66.7%) |  | 5200~6600 (100%) | 3900~5100 (100%) | 7000~22500 (100%) |  |  |  |  |  | [12] |
| Surface water (river) | Rio de Janeiro, Brazil | 2017~2018 | 16 | ng/L | 89~587 (100%) | ＜9~473 (87.5%) |  |  |  |  |  |  |  |  | [13] |
| Surface water | Tianjin, China | 2013 | 18 | ng/L | n.d.~6019 (16.7%) |  | n.d.~41083 (16.7%) |  | n.d.~13005 (83.3%) | n.d.~2013 (11.1%) |  |  |  |  | [14] |
| Surface water (lagoon and channel) | Rio de Janeiro, Brazil | 2017~2018 | 16 | ng/L | ＜10~302 (56.25%) | ＜9~2500 (50%) |  |  |  |  |  |  |  |  | [13] |
| Surface water (reservoir) | Taoyuan, Taiwan, China | — | 4 | ng/L | n.d.~6300 (50%) |  | 2000~8300 (100%) | 5200~7100 (100%) | 10800~41100 (100%) |  |  |  |  |  | [12] |
| Bottom water | Pearl River estuary, China | 2021 | 8 | ng/L | 32~106 (100%) |  | n.d.~48 (87.5%) |  | 8.3~73 (100%) |  |  |  |  |  | [2] |
| Bottom water | Pearl River estuary, China | 2022 | Dry season: 15 Wet season: 14 | ng/L | Dry season: 16.8~105 (100%) Wet season: 8.7~206 (100%) |  | Dry season: 18.5~178 (100%) Wet season: 33.3~1676 (100%) | Dry season: ＜0.09~0.4 (66.7%) Wet season: 0.1~0.95 (71.4%) | Dry season: 4.82~44.3 (100%) Wet season: 7.28~38.8 (100%) |  |  |  |  |  | [5] |
| Groundwater | Guangzhou, China | 2020 | 43 | ng/L | n.d.~6187 (227, 90.7%) |  |  | n.d.~119 (6.38, 97.7%) | n.d.~7446 (182, 72.1%) | n.d.~28.8 (3.28, 16.3%) | n.d.~0.81 (0.19, 51.2%) |  |  |  | [1] |
| Sewage ditch water | Tianjin, China | 2013 | 2 | ng/L | n.d.~30867 (50%) |  | n.d.~18185 (50%) |  | 1753~31765 (100%) | n.d.~7904 (50%) |  |  |  |  | [14] |
| Sewage ditch water | Taoyuan, Taiwan, China | — | 4 | ng/L |  |  |  | n.d.~5300 (100%) | 6500~10700 (100%) |  |  |  |  |  | [12] |
| Sewage ditch water | Kerala, India | 2008 | 1 | ng/L | 2500000 |  | 800000 |  | 650000 | 28000 |  |  |  |  | [11] |
| Seawater | Yangtze River estuary and its adjacent area, China | — | 11 | ng/L | n.d.~7.56 (90.9%) |  | n.d.~5.09 (81.8%) |  | n.d.~6.51 (81.8%) |  |  |  |  |  | [15] |
| Surface seawater | Yangtze River estuary and its adjacent area, China | 2021~2022 | Winter-Spring: 32 Summer: 36 Autumn: 44 | ng/L | Winter-Spring: ＜0.12~12.19 (4.66, 96.9%) Summer: ＜0.12~36.19 (5.48, 91.7%) Autumn: ＜0.12~21.76 (6.53, 91.1%) |  | Winter-Spring: ＜0.03~8.32 (2.35, 87.5%) Summer: ＜0.03~16.48 (2.19, 88.9%) Autumn: ＜0.03~8.15 (1.28, 97.8%) |  | Winter-Spring: ＜0.08~6.51 (1.81, 81.3%) Summer: ＜0.08~24.32 (3.83, 91.7%) Autumn: ＜0.08~3.32 (0.66, 88.9%) |  |  |  |  |  | [16] |
| Surface seawater | South China Sea | 2022 | 39 | ng/L | 0.53~6.81 (4.11, 100%) |  | 0.4~5.69 (2.7, 100%) |  | 0.59~9.36 (4.25, 100%) |  |  |  |  |  | [17] |
| Surface and bottom seawater | Greenland Sea | 2021 | 22 | ng/L | n.d.~907 (355, 73%) |  | n.d.~273 (114, 64%) | 0.1~1 (0.3, 100%) | n.d.~45 (34, 68%) | n.d.~22 (15, 50%) | n.d.~4 (0.5, 95%) |  |  | 0.2~1043 (191, 100%) | [18] |
| Suspended particulate matter | Yangtze River estuary and its adjacent area, China | 2021~2022 | Summer: 18 Autumn: 44 | ng/L | Summer: 0.82~13.18 (3.82, 100%) Autumn: ＜0.02~15.33 (1.11, 97.8%) |  | Summer: 0.16~8.76 (2.09, 100%) Autumn: 0.04~3.24 (0.69, 100%) |  | Summer: ＜0.01~2 (0.87, 55.6%) Autumn: ＜0.01~2.46 (0.36, 93.3%) |  |  |  |  |  | [16] |
| Suspended particulate matter | South China Sea | 2022 | 36 | ng/g dw | 20~210 (100, 100%) |  | 10~370 (100, 100%) |  | 60~450 (190, 100%) |  |  |  |  |  | [17] |
| Surface suspended particulate matter | Pearl River estuary, China | 2022 | Dry season: 18 Wet season: 18 | ng/g | Dry season: 1368~1446 (16.7%) Wet season: 344~5149 (88.9%) |  | Dry season: 32.5~541 (88.9%) Wet season: 19.6~162 (83.3%) | Dry season: 0.92~4.82 (22.2%) Wet season: ＜0.09~9.62 (55.6%) | Dry season: 223~718 (100%) Wet season: 3.22~290 (100%) |  |  |  |  |  | [5] |
| Bottom suspended particulate matter | Pearl River estuary, China | 2022 | Dry season: 15 Wet season: 14 | ng/g | Dry season: 492~3256 (53.3%) Wet season: 259~2248 (85.7%) |  | Dry season: 43~217 (93.3%) Wet season: 12.9~132 (92.9%) | Dry season: 1.52~1.79 (13.3%) Wet season: 0.39~3.49 (42.9%) | Dry season: 165~1243 (100%) Wet season: 17.9~197 (100%) |  |  |  |  |  | [5] |
| Surface sediment | North River, Pearl River basin, China | 2018 | Dry season: 13 Wet season: 13 | ng/g | Dry season: n.d.~9.39 (8%) Wet season: n.d.~21.4 (85%) | Dry season: n.d.~14.9 (62%) Wet season: 9.04~23.5 (100%) | Dry season: 3.48~12.15 (100%) Wet season: 2.82~10.2 (100%) |  | Dry season: 0.63~3.5 (100%) Wet season: n.d.~30.7 (15%) | Dry season: n.d.~8.92 (8%) Wet season: n.d.~32.7 (15%) |  |  |  |  | [3] |
| Surface sediment | Pearl River estuary, China | 2021 | 8 | ng/g | n.d.~33 (37.5%) |  | n.d.~2.6 (25%) |  | n.d.~1.8 (75%) |  |  |  |  |  | [2] |
| Surface sediment | West River, Pearl River basin, China | 2018 | Dry season: 22 Wet season: 22 | ng/g | Dry season: n.d.~3.39 (5%) Wet season: n.d.~6.18 (77%) | Dry season: n.d.~12.6 (64%) Wet season: n.d.~11.3 (45%) | Dry season: 2.9~12.9 (100%) Wet season: 2.69~4.98 (100%) |  | Dry season: 0.55~4.32 (100%) Wet season: n.d. | Dry season: n.d.~3.72 (5%) Wet season: n.d.~12.1 (5%) |  |  |  |  | [3] |
| Surface sediment | Yangtze River estuary and its adjacent area, China | — | 11 | ng/g dw | n.d.~3.26 (81.8%) |  | n.d.~6.3 (90.9%) |  | 0.09~4.34 (100%) |  |  |  |  |  | [15] |
| Surface sediment | Pearl River estuary, China | 2022 | Dry season: 18 Wet season: 18 | ng/g | Dry season: ＜3.53 (27.8%) Wet season: ＜3.53 (22.2%) |  | Dry season: ＜1.38~6.25 (27.8%) Wet season: ＜1.38~2.6 (55.6%) | Dry season: ＜0.09~0.39 (22.2%) Wet season: 0.11 (5.56%) | Dry season: 0.43~1.91 (100%) Wet season: ＜0.24~2.02 (100%) |  |  |  |  |  | [5] |
| Surface sediment | Rhode Island, USA | 1995 | 12 | ng/g dw |  |  | ＜20~31 (8.3%) |  |  |  |  |  | ＜1~1.31 (16.7%) |  | [19] |
| Surface sediment | Yangtze River estuary and its adjacent area, China | 2021~2022 | Winter-Spring: 29 | ng/g dw | Winter-Spring: ＜0.21~1.54 (0.52, 79.3%) |  | Winter-Spring: ＜0.13~1.44 (0.34, 96.5%) |  | Winter-Spring: ＜0.03~1.57 (0.47, 89.6%) |  |  |  |  |  | [16] |
| Surface sediment | South China Sea | 2021 | 15 | ng/g dw | 0.16~0.45 (0.23, 100%) |  | 0.04~0.72 (0.24, 100%) |  | 0.26~0.65 (0.44, 100%) |  |  |  |  |  | [17] |
| Runoff sediment | Aachen, Germany | 2018 | 5 | ng/g dw |  |  | 134.9~815.9 (100%) |  | 281.6~2510.4 (100%) |  |  |  |  |  | [20] |
| Runoff sediment | Oslo, Norway | 2016 | 3 | ng/g dw |  |  | ＜MDL~1000 (66.7%) |  |  |  |  |  |  |  | [21] |
| Runoff (road tunnel wash water) | Oslo, Norway | 2022 | 9 | ng/L |  |  | 2986~13914 (8233, 100%) | 71~394 (285, 100%) | 140~966 (492, 100%) |  |  |  |  |  | [22] |
| Runoff (riverine) | Pearl River Delta, China | 2005~2006 | 80 | ng/L | 0.48~3190 (100%) |  |  |  | 1.35~413 (100%) |  |  |  | n.d.~1.81 (45%) |  | [23] |
| Runoff (courtyard) | Two cities, China | 2015 | 12 | ng/L | 348~2782 (945, 100%) |  | 662~2875 (1265, 100%) | 6.63~916 (148, 100%) | 142~1349 (352, 100%) |  |  |  |  |  | [6] |
| Runoff (road) | Two cities, China | 2015 | 16 | ng/L | 745~15430 (4610, 100%) |  | 941~9292 (2984, 100%) | 69.9~13260 (2167, 100%) | 180~4170 (1258, 100%) |  |  |  |  |  | [6] |
| Runoff (farmland) | Two cities, China | 2015 | 6 | ng/L | 179~981 (608, 100%) | 6.5~685 (298, 100%) | 229~618 (489, 100%) | 1.63~38.6 (10.6, 100%) | 58.7~177 (108, 100%) |  |  |  |  |  | [6] |
| Runoff (highway) | Five cities, China | 2022~2023 | 8 | ng/L | 26~14470 (4033, 100%) | n.d.~636 (196, 64%) | 73.1~34430 (3607, 100%) | 18.8~1441 (356, 100%) | 17~664 (207, 100%) | n.d.~843 (172, 93%) |  |  |  |  | [24] |
| Runoff (business district) | Five cities, China | 2022~2023 | 7 | ng/L | 19.9~16880 (4868, 100%) | n.d.~1620 (360, 60%) | 104~2784 (798, 100%) | 7.1~1230 (290, 100%) | 14.2~873 (395, 100%) | 12.1~2770 (358, 100%) |  |  |  |  | [24] |
| Runoff (urban village) | Five cities, China | 2022~2023 | 7 | ng/L | n.d.~17960 (3355, 91%) | n.d.~3760 (450, 82%) | 120~4102 (1027, 100%) | n.d.~2164 (366, 91%) | 21.9~662 (301, 100%) | 75~655 (278, 100%) |  |  |  |  | [24] |
| Runoff (green space) | Five cities, China | 2022~2023 | 4 | ng/L | 26.8~7300 (2280, 100%) | n.d.~430 (150, 75%) | 67.2~295 (186, 100%) | 3.3~176 (51.3, 100%) | 11.2~398 (134, 100%) | 39.4~1744 (567, 100%) |  |  |  |  | [24] |
| Runoff (residential area) | Five cities, China | 2022~2023 | 5 | ng/L | 113~9103 (2765, 100%) | n.d.~1827 (376, 60%) | 126~3234 (691, 100%) | 6.2~308 (82.5, 100%) | 26.7~278 (159, 100%) | 63.3~1253 (485, 100%) |  |  |  |  | [24] |
| Runoff (tourist area) | Five cities, China | 2022~2023 | 3 | ng/L | 1910~4124 (3359, 100%) |  | 309~1615 (850, 100%) | 229~1032 (624, 100%) | 192~366 (304, 100%) | 48.3~412 (212, 100%) |  |  |  |  | [24] |
| Runoff (industrial area) | Five cities, China | 2022~2023 | 5 | ng/L | 19.1~1639 (622, 100%) | n.d.~184 (36.8, 20%) | 101~1350 (404, 100%) | 10.1~332 (117, 100%) | 4.1~368 (162, 100%) | 6.5~796 (228, 100%) |  |  |  |  | [24] |
| Runoff (port area) | Five cities, China | 2022~2023 | 3 | ng/L | 28.8~818 (306, 100%) | 3.6~4309 (1449, 100%) | 127~457 (258, 100%) | 21.2~178 (74.1, 100%) | 5.9~151 (61.7, 100%) | 55.5~486 (278, 100%) |  |  |  |  | [24] |
| Runoff | Aachen, Germany | 2018 | 5 | ng/L |  |  | 128.7~303.2 (100%) |  | 1544~8805.2 (100%) | n.d.~185.5 (80%) |  |  | 23.1~44.3 (100%) |  | [20] |
| Runoff | Rhode Island, USA | 1995 | 11 | ng/L | 378~1210 (100%) |  | 721~6910 (100%) |  |  |  |  |  | 154~278 (100%) |  | [19] |
| Runoff (dissolved phase) | Aachen, Germany | 2018 | 5 | ng/g silicone | 410.5~1269 (100%) |  | n.d.~608.1 (60%) |  | 963.7~2822.6 (100%) | 6.5~24.8 (100%) |  |  | n.d.~61.9 (80%) |  | [20] |
| Stormwater | Guangzhou, China | 2021 | 10 | ng/L | 11.7~70.8 (38.5, 100%) |  |  | n.d.~6.72 (2.29, 50%) | 1.21~82.4 (28.6, 100%) | n.d.~12 (5.01, 50%) |  |  |  |  | [1] |
| Raw drinking water | Korea | 2019~2021 | 120 | ng/L | ＜0.9~168 (56.7, 100%) |  | n.d.~456 (45.4, 98%) | n.d.~0.359 (0.021, 6%) | n.d.~11.4 (2.98, 95%) | n.d.~7.52 (1.8, 73%) |  |  |  | 49.3~631 (117, 100%) | [25] |
| Raw drinking water | Korea | 2023 | 126 | ng/L | n.d.~493 (52.2, 78.6%) |  | n.d.~151 (3.92, 10.3%) |  | n.d.~28.7 (1.3, 32.5%) | n.d.~8.79 (0.537, 16.7%) |  |  |  |  | [26] |
| Drinking water | Fifty-one cities, China | 2014 | 86 | ng/L | 32.5~1100 (100%) |  | ＜2.3~203 (59.3%) | ＜1.3~4.9 (8.1%) |  |  |  |  |  | 40.1~1310 (100%) | [27] |
| Drinking water | Korea | 2023 | 140 | ng/L | n.d.~277 (24.4, 57.1%) |  | n.d.~19.2 (0.398, 4.3%) |  | n.d.~10.6 (0.414, 10%) | n.d.~5.9 (0.316, 16.4%) |  |  |  |  | [26] |
| Drinking water | Korea | 2019~2021 | 120 | ng/L | 18.7~134 (50.9, 100%) |  | n.d.~87.9 (13.7, 77%) |  | n.d.~4.05 (0.823, 77%) | n.d.~6.55 (1.05, 53%) |  |  |  | 18.7~149 (66.5, 100%) | [25] |
| Bottled water | Korea | 2019~2021 | 11 | ng/L | 31.3~97.6 (41.9, 100%) |  | 2.74~8.63 (4.89, 100%) |  | 0.315~1.53 (0.784, 100%) | 0.662~1.43 (0.799, 100%) |  |  |  | 37.4~104 (48.4, 100%) | [25] |
| Tea beverage | Chung-Li city, Taiwan, China | — | 10 | ng/L |  |  | n.d.~13600 | n.d.~＜5000 |  |  |  |  |  |  | [28] |
| Wine | Santa Maria Imbaro, Italy | — | 12 | ng/L | 240~1090 (100%) |  |  |  |  |  |  |  |  |  | [29] |
| Wine | Italy | 2006 | 80 | ng/L | 1000~14100 (5200) |  |  |  |  |  |  |  |  |  | [30] |
| Sparkling Wine | Trentino-South Tyrol, Italy | 2007, 2009 | 15 | ng/L | 3200~5580 (100%) |  |  |  |  |  |  |  |  |  | [31] |
| Wastewater (particulate matter) | Athens, Greece | 2012 | 4 | ng/L | 89~210 (100%) |  | 16~86 (100%) |  | 18~51 (100%) |  |  |  |  |  | [32] |
| Wastewater (dissolved phase) | Athens, Greece | 2012 | 10 | ng/L | n.d.~14010 (60%) |  | 102~30224 (100%) | n.d.~248 (20%) | n.d.~1887 (60%) |  |  |  |  |  | [32] |
| Household wastewater | Berlin, Germany | 2004 | 8 | ng/L | (640, 100%) |  | (350, 13%) |  | (90, 38%) |  |  |  |  |  | [33] |
| Urban wastewater influent | Northwest Spain | — | 6 | ng/L | (830) |  | (150) |  |  |  |  |  |  |  | [34] |
| Industrial wastewater influent | Northwest Spain | — | 3 | ng/L | (170) | (580) | (10900) |  |  |  |  |  |  |  | [34] |
| Tannery wastewater influent | Berlin, Germany | 2000 | 5 | ng/L | (11600) | (746900) |  | (240) | (13500) |  |  |  |  |  | [35] |
| Wastewater influent | Three cities, China | 2011~2013 | 10 | ng/L | 200~3310 (830) |  |  |  | n.d.~2780 (580) |  |  |  |  |  | [36] |
| Wastewater influent | North and Southwest India | 2012 | 5 | ng/L | 3310~116000 (49300, 100%) |  | 293~579 (423, 100%) |  | 217~2185 (999, 100%) |  |  |  |  | 3800~118800 (50800, 100%) | [37] |
| Wastewater influent | Berlin, Germany | 2002, 2003 | 29 | ng/L | (800, 97%) | (110, 59%) | (330, 17%) |  | (310, 100%) |  |  |  |  |  | [33] |
| Wastewater influent | Tuebingen, Germany | 2009 | 6 | ng/L | 577~2669 (100%) |  |  |  |  |  |  |  |  |  | [8] |
| Wastewater influent | Albany, New York, USA | 2023 | 8 | ng/L | 92.3~146 (109, 75%) |  | 8.3~81.5 (28.2, 88%) | 1.23~11.6 (3.59, 100%) | 42.9~166 (78.5, 100%) |  |  |  |  |  | [38] |
| Wastewater influent | Athens, Greece | 2010~2011 | 14 | ng/L | 528~1452 (1096, 100%) |  | 256~908 (503, 100%) | n.d.~18 (n.d., 14%) | 219~4522 (719, 100%) |  |  |  |  |  | [39] |
| Wastewater influent | Albany, New York, USA | 2013~2015 | WWTP_A_: 16 WWTP_B_: 16 | ng/L | WWTP_A_: 1580~23900 (8440, 100%) WWTP_B_: 1020~25800 (7300, 100%) |  | WWTP_A_: 329~354 (12.5%) WWTP_B_: ＜0.23~280 (0.98, 25%) |  | WWTP_A_: 36.6~906 (207, 100%) WWTP_B_: ＜0.06~581 (110, 93.75%) |  |  |  |  | WWTP_A_: 1660~24600 (8690, 100%) WWTP_B_: 1020~26400 (7550, 100%) | [40] |
| Wastewater primary effluent | Albany, New York, USA | 2013~2015 | WWTP_A_: 16 WWTP_B_: 16 | ng/L | WWTP_A_: 189~22400 (6230, 100%) WWTP_B_: 1290~20500 (6260, 100%) |  | WWTP_A_: 54.2~662 (12.5%) WWTP_B_: 308 (6.25%) |  | WWTP_A_: ＜0.06~1090 (140, 93.75%) WWTP_B_: ＜0.06~533 (114, 93.75%) |  |  |  |  | WWTP_A_: 241~22900 (6530, 100%) WWTP_B_: 1380~21100 (6470, 100%) | [40] |
| Wastewater primary effluent | Albany, New York, USA | 2023 | 4 | ng/L | 17.2~120 (79.6, 75%) |  | 8.35~12.8 (10.8, 75%) | 2.08~9.9 (5.22, 100%) | 24.1~36.2 (30.2, 100%) |  |  |  |  |  | [38] |
| Wastewater secondary effluent | Three cities, China | 2011~2013 | 14 | ng/L | n.d.~1780 (340) |  |  |  | 10~320 (140) |  |  |  |  |  | [36] |
| Wastewater final effluent | Three cities, China | 2011~2013 | 8 | ng/L | n.d.~270 (80) |  |  |  | n.d.~200 (110) |  |  |  |  |  | [36] |
| Wastewater final effluent | Albany, New York, USA | 2013~2015 | WWTP_A_: 16 WWTP_B_: 16 | ng/L | WWTP_A_: 1230~17800 (6890, 100%) WWTP_B_: 1770~21200 (6470, 100%) |  | WWTP_A_: ＜0.23~653 (1.5, 31.25%) WWTP_B_: ＜0.23~224 (0.87, 25%) |  | WWTP_A_: ＜0.06~617 (87.1, 87.5%) WWTP_B_: ＜0.06~501 (67.7, 87.5%) |  |  |  |  | WWTP_A_: 1270~19000 (7150, 100%) WWTP_B_: 1770~21500 (6650, 100%) | [40] |
| Wastewater final effluent | Albany, New York, USA | 2023 | 8 | ng/L | 75~204 (134, 75%) |  | 47.4~169 (71.8, 88%) | 2.35~10.3 (4.49, 100%) | 14.1~45.8 (31.1, 100%) |  |  |  |  |  | [38] |
| Tannery wastewater effluent | Berlin, Germany | 2000 | 5 | ng/L | (7500) | (1100) | (3900) | (390) | (3500) |  |  |  |  |  | [35] |
| Urban wastewater effluent | Northwest Spain | — | 6 | ng/L | (740) | (260) | (440) |  | (60) |  |  |  |  |  | [34] |
| Industrial wastewater effluent | Northwest Spain | — | 3 | ng/L |  | (340) | (100) |  |  |  |  |  |  |  | [34] |
| Wastewater effluent | Athens, Greece | 2010~2011 | 14 | ng/L | 89~616 (254, 100%) |  | 94~514 (251, 100%) | n.d.~31 (＜14, 21%) | 36~368 (112, 100%) |  |  |  |  |  | [39] |
| Wastewater effluent | North and Southwest India | 2012 | 5 | ng/L | 1330~43400 (19500, 100%) |  | 38.7~305 (145, 100%) |  | 75.1~1381 (513, 100%) |  |  |  |  | 1520~45000 (20200, 100%) | [37] |
| Wastewater effluent | Berlin, Germany | 2002, 2003 | 30 | ng/L | (300, 97%) | (10, 17%) | (200, 27%) |  | (400, 100%) |  |  |  |  |  | [33] |
| Wastewater effluent | Beijing, China | 2003 | 4 | ng/L | (2260, 100%) | (40, 25%) | (1540, 100%) |  | (550, 100%) |  |  |  |  |  | [33] |
| Wastewater effluent | Tuebingen, Germany | 2009 | 6 | ng/L | n.d.~497 (50%) |  |  |  |  |  |  |  |  |  | [8] |
| Sludge | Catalonia, Spain | — | 10 | ng/g dw | n.d.~26.2 (70%) |  | ＜0.5~255.4 (100%) | n.d.~17 (50%) |  |  |  |  |  |  | [41] |
| Sludge | Albany, New York, USA | 2013~2015 | WWTP_A_: 13 WWTP_B_: 15 | ng/g dw | WWTP_A_: ＜2.44~7180 (228, 84.6%) WWTP_B_: 65.9~3450 (750, 100%) |  | WWTP_A_: ＜3.74~270 (59.5, 92.3%) WWTP_B_: ＜3.74~2910 (53.2, 80%) |  | WWTP_A_: 39.4~1430 (162, 100%) WWTP_B_: 21.9~218 (83.5, 100%) |  |  |  |  | WWTP_A_: 142~7780 (764, 100%) WWTP_B_: 185~5820 (1090, 100%) | [40] |
| Sludge | Koblenz, Germany | 2008 | 4 | ng/g dw | (265) |  | (307) |  | (157) |  |  |  | (5.3) |  | [42] |
| Sludge | North and Southwest India | 2012 | 5 | ng/g dw | 14800~85700 (50200, 100%) |  | ＜LOQ~586 (173.8, 60%) |  | 105.2~1950 (826.3, 100%) |  |  |  |  | 14900~88200 (51200, 100%) | [37] |
| Sludge | Albany, New York, USA | 2023 | 3 | ng/g dw | ＜LOD~22.2 (＜LOD, 33%) |  | ＜LOD~173 (＜LOD, 67%) | ＜LOD~9.9 (＜LOD, 33%) | 1.98~19.4 (7.88, 100%) |  |  |  |  |  | [38] |
| Sludge | Catalonia, Spain | — | 10 | ng/g dw |  |  | ＜1~181.2 (60%) |  | n.d.~39.8 (20%) |  |  |  |  |  | [43] |
| Dewatered sludge | Athens, Greece | 2010~2011 | 14 | ng/g dw | n.d.~174 (86, 93%) |  | 33~312 (189, 100%) |  | 23~77 (52, 100%) |  |  |  |  |  | [39] |
| Dewatered sludge | Athens, Greece | 2012 | 2 | ng/g dw | 88~174 (100%) |  | 33~74 (100%) |  | 61 (100%) |  |  |  |  |  | [32] |
| Clothing textile | Stockholm, Sweden | — | 24 | ng/g | n.d.~231 (91.7%) | n.d.~2100 (4.2%) |  |  |  |  |  |  |  |  | [44] |
| Clothing textile | Stockholm, Sweden | 2011~2012 | 26 | ng/g | n.d.~50900 (88.5%) |  |  |  | n.d.~1700 (53.8%) |  |  |  |  |  | [45] |
| Clothing textile | Switzerland | 2011 | 1 | ng/g | (8640, 100%) |  |  |  | (738, 100%) |  |  |  |  |  | [46] |
| Clothing textile | Albany, New York, USA | 2016 | 79 | ng/g | ＜2.9~1120 (101, 86%) |  | ＜1.6~95.7 (9.36, 19%) |  | ＜1.7~47.3 (6.32, 54%) |  |  |  |  |  | [47] |
| Outdoor air (Vapor phase) | Constantí, Tarragona, Spain | 2021~2022 | 25 | pg/m^3^ | Winter: n.d.~1509 (853, 86%) Summer: 418~2958 (1217, 100%) |  | Winter: n.d.~2621 (301, 14%) |  | Winter: n.d.~121 (9, 7%) Summer: n.d.~2968 (982, 82%) |  |  | Winter: n.d.~164 (19, 21%) Summer: ＜0.9 (＜0.9, 100%) |  |  | [48] |
| Outdoor air (Vapor phase) | Nine cities, China | 2018 | 126 | pg/m^3^ | 212~6170 (988, 100%) |  | n.d.~298000 (48500, 97%) | n.d.~36.1 (5.11, 78%) | 30.2~414 (144, 100%) | n.d.~167 (20.5, 99%) | n.d.~7.05 (0.63, 31%) |  |  |  | [49] |
| Urban PM_10_ | Saxony, Germany | 2023 | 23 | pg/m^3^ | n.d.~28.46 (24.11, 65%) |  | 1.7~38.24 (12.77, 100%) | 1.48~6.27 (2.95, 100%) |  |  |  |  | 16.38~118.93 (50.75, 100%) |  | [50] |
| Rural PM_10_ | Saxony, Germany | 2023 | 5 | pg/m^3^ |  |  | 2.29~31.96 (100%) | 1.48~3.46 (100%) |  |  |  |  | 20.64~83.97 (100%) |  | [50] |
| Outdoor air (PM_10_) | Constantí, Tarragona, Spain | 2017 | 10 | pg/m^3^ | 40~950 (350, 100%) |  | 90~7600 (1200, 100%) | n.d.~380 (110, 40%) | n.d.~100 (20, 40%) |  |  | n.d.~＜7.2 (＜7.2, 90%) |  | 170~8900 (1700, 74%) | [51] |
| Outdoor air (PM_10_) | Tarragona harbour, Spain | 2017 | 10 | pg/m^3^ | ＜5.8~200 (40, 100%) |  | n.d.~1500 (610, 70%) | n.d.~1100 (190, 20%) | n.d.~690 (110, 60%) |  |  | n.d.~＜7.2 (＜7.2, 20%) |  | 9~3300 (960, 54%) | [51] |
| Outdoor air (PM_10_) | Constantí, Tarragona, Spain | 2021~2022 | 25 | pg/m^3^ | Winter: 45~2611 (624, 100%) Summer: 56~3294 (776, 100%) |  | Winter: ＜147 (＜147, 100%) Summer: n.d.~380 (151, 73%) | Summer: ＜0.6 (＜0.6, 100%) | Winter: n.d.~141 (30, 36%) Summer: n.d.~44 (15, 55%) |  |  | Winter: ＜0.9~11 (1, 64%) Summer: ＜0.9 (＜0.9, 100%) |  |  | [48] |
| Urban PM_10-2.5_ | Saxony, Germany | 2023 | 10 | pg/m^3^ |  |  | n.d.~26.8 (90%) | n.d.~1.8 (90%) |  |  |  |  | n.d.~32.93 (60%) |  | [50] |
| Urban PM_2.5_ | Saxony, Germany | 2023 | 10 | pg/m^3^ |  |  | 9.09~19.92 (100%) | 1.89~4.93 (100%) |  |  |  |  | 32.35~136.53 (100%) |  | [50] |
| Outdoor air (PM_2.5_) | Nine cities, China | 2018 | 126 | pg/m^3^ | n.d.~1200 (307, 99%) |  | n.d.~1130 (153, 99%) | n.d.~33.9 (5.4, 92%) | n.d.~15.9 (2.13, 79%) | n.d.~4.49 (0.49, 29%) |  |  |  |  | [49] |
| Outdoor air (PM_2.5_) | Guangzhou, China | 2018~2019 | 8 | pg/m^3^ | 18.4~47.1 (32.6, 100%) |  | 206~557 (416, 100%) | 22.8~45.9 (31.1, 100%) | 10.8~29.1 (19.8, 100%) |  |  | 4.91~16.9 (8.99, 100%) |  |  | [52] |
| Outdoor air (PM_2.5_) | Shanghai, China | 2018~2019 | 8 | pg/m^3^ | 23.1~78.4 (46.8, 100%) |  | 132~477 (263, 100%) | 20.1~29.2 (24.1, 100%) | 9.38~28.2 (18.1, 100%) |  |  | n.d.~6.41 (3.45, 87.5%) |  |  | [52] |
| Outdoor air (PM_2.5_) | Taiyuan, China | 2018~2019 | 10 | pg/m^3^ | 36.6~78.4 (55.9, 100%) |  | 66.8~430 (164, 100%) | 24.2~44.2 (32.6, 100%) | 14~39.5 (25.4, 100%) |  |  | 5.45~19.3 (11, 100%) |  |  | [52] |
| Outdoor aerosol | Mestre, Venezia, Italy | 2023 | 9 | pg/m^3^ | 48~927 (309) | 46~1000 (331) | 24~201 (88) | 1~3 (2) | 55~461 (186) | n.d.~20 (6) | n.d.~3 (1) |  |  |  | [53] |
| Indoor air | Albany, New York, USA | 2014 | 81 | pg/m^3^ | 2940~1703000 (26900, 100%) |  | 180~872000 (2470, 100%) | ＜200~22200 (90, 96.3%) | ＜200~115000 (320, 92.6%) |  |  |  |  | 4360~2229000 (32700, 100%) | [54] |
| Indoor aerosol (TSP) | Mestre, Venezia, Italy | 2023 | 4 | pg/m^3^ | 100~800 (500, 100%) | 200~1100 (800, 100%) | 200~1000 (600, 100%) | 2~5 (4) | 200~500 (300, 100%) | 10~30 (20, 100%) | 0.4~1.4 (1) |  |  |  | [55] |
| Indoor aerosol (PM_10_) | Mestre, Venezia, Italy | 2023 | 4 | pg/m^3^ | 100~500 (300, 100%) | 200~800 (600, 100%) | 100~800 (500, 100%) | 2~5 (4) | 100~300 (200, 100%) | 4~30 (20, 100%) | 0.4~1.4 (0.8) |  |  |  | [55] |
| Indoor aerosol (PM_1_) | Mestre, Venezia, Italy | 2023 | 4 | pg/m^3^ | 30~300 (200, 100%) | 100~700 (500, 100%) | 100~700 (400, 100%) | 2~5 (4) | 100~200 (200, 100%) | 2~20 (10, 100%) | 0.4~1.4 (0.8) |  |  |  | [55] |
| Road dust | Hong Kong, China | 2023 | 24 | ng/g | 6.8~467 (123, 100%) | ＜LOQ~263 (45.9, 79%) | 31.6~1480 (228, 100%) | 0.789~65.6 (15.3, 100%) | 2.51~431 (84.4, 100%) |  |  |  | ＜LOQ~2.03 (0.265, 46%) |  | [56] |
| Road dust | Berlin, Germany | 2023 | 43 | ng/g | ＜LOQ~548 (82.9, 95%) | ＜LOQ~393 (34.5, 58%) | ＜LOQ~436 (108, 98%) | ＜LOQ~130 (10.9, 98%) | ＜LOQ~63.5 (5.66, 70%) |  |  |  | ＜LOQ~17.9 (1.05, 47%) |  | [56] |
| Road dust | Rhode Island, USA | 1995 | 2 | ng/g | 78.7~149 (100%) |  | 24.6~90.2 (100%) |  |  |  |  |  | 1.68~2.45 (100%) |  | [19] |
| Road dust | Trondheim, Norway | 2017 | 32 | ng/L | Non-studded season: n.d.~66.4 (35.5, 62.5%) Studded season: n.d.~203 (127, 56.25%) | Non-studded season: 6.79~19 (12.6, 100%) Studded season: 1.72~19.4 (9.25, 100%) | Non-studded season: 26.6~522 (232, 100%) Studded season: 245~1404 (729, 100%) | Non-studded season: 27~162 (52, 100%) Studded season: 26.9~134 (59.1, 100%) | Non-studded season: 16.8~116 (41.2, 100%) Studded season: 77.4~195 (126, 100%) |  |  |  | Non-studded season: 0.06~9.21 (1.92, 100%) Studded season: 1.72~6.61 (3.47, 100%) | Non-studded season: 93.4~801 (361, 100%) Studded season: 360~1903 (1006, 100%) | [57] |
| Road dust | Tianjin, China | 2016 | 35 | ng/g | TSP (＜75 μm): 30~370 (100%) PM_10_: ＜LOQ~27460 (97%) PM_2.5_: 2660~55900 (100%) | TSP: ＜LOQ~50 (58%) PM_10_: ＜LOQ~2190 (94%) PM_2.5_: 360~5430 (92%) | TSP: 180~970 (100%) PM_10_: ＜LOQ~23080 (97%) PM_2.5_: 4400~31370 (100%) | TSP: 40~220 (100%) PM_10_: ＜LOQ~800 (89%) PM_2.5_: ＜LOQ~7260 (94%) | TSP: ＜LOQ~20 (58%) PM_10_: ＜LOQ~1440 (97%) PM_2.5_: ＜LOQ~9030 (94%) |  |  |  |  |  | [58] |
| Road dust | Guangzhou, China | 2022~2023 | 28 | ng/g |  | ＜2.22~90.7 (79%) | 4.69~28.6 (100%) | 0.301~3.55 (100%) | 26.1~133 (100%) | ＜8.37~159 (36%) |  |  |  |  | [59] |
| Road dust | Guangzhou, China | 2021 | 60 | ng/g | ＜75.5~8599 (98%) |  | ＜120~4026 (87%) |  | ＜96.5~3317 (98%) |  |  | 298~12798 (100%) | <0.4~29.6 (71%) |  | [60] |
| Indoor dust | Tarragona, Spain | 2020~2021 | 16 | ng/g | ＜170~3230 (700, 100%) |  |  |  |  |  |  |  |  |  | [61] |
| Indoor dust | Albany, New York, USA | 2006, 2010 | 40 | ng/g | ＜0.5~546 (27.5%) |  | 251~13600 (100%) | ＜0.5~12.4 (12.5%) | ＜1~1340 (80%) |  | ＜1~757 (70%) |  |  | 277~13800 (100%) | [62] |
| Indoor dust | Six cities, China | 2010 | 55 | ng/g | ＜0.5~2780 (98.2%) |  | 3.85~4020 (100%) | ＜0.5~6.16 (9.1%) | ＜1~264 (92.7%) |  | ＜1~26.2 (92.7%) |  |  | 119~6540 (100%) | [62] |
| Indoor dust | Five cities, Japan | 2012 | 22 | ng/g | ＜0.5~43.2 (13.6%) |  | 88.9~5550 (100%) |  | ＜1~938 (86.4%) |  | ＜1~49.8 (95.5%) |  |  | 158~5900 (100%) | [62] |
| Indoor dust | Two cities, Korea | 2012 | 41 | ng/g | ＜0.5~392 (61%) |  | 207~9060 (100%) | ＜0.5~13 (31.7%) | ＜1~772 (75.6%) |  | ＜1~63 (82.9%) |  |  | 222~9420 (100%) | [62] |
| Indoor dust | Two cities, China | 2018~2019 | 79 | ng/g | ＜0.671~2510 (60.2, 94.9%) |  | 3.87~2590 (38.5, 100%) | ＜0.354~865 (1.33, 84.8%) | ＜0.237~275 (8.43, 97.5%) | ＜0.511~13.6 (0.558, 27.8%) | ＜0.799~1.17 (0.573, 2.53%) |  |  | 12.6~5430 (148, 100%) | [63] |
| Indoor dust | Qingyuan, China | 2016~2017 | 25 | ng/g | ＜3.5~458 (117, 96%) | ＜1.2~242 (48.8, 92%) | 158~2530 (479, 100%) | ＜0.6~144 (46.9, 96%) | ＜3~185 (21.8, 76%) | ＜2~123 (11.3, 52%) |  |  |  |  | [64] |
| Indoor dust | Guangzhou, China | 2016~2017 | 24 | ng/g | ＜3.5~1560 (232, 96%) | 42.3~283 (116, 100%) | 211~1640 (500, 100%) | ＜0.6~123 (8.67, 58%) | ＜3~231 (7.81, 63%) | ＜2~29.8 (4.63, 38%) |  |  |  |  | [64] |
| Household dust | Guangzhou, China | 2022~2023 | 37 | ng/g |  | ＜2.22~845 (76%) | ＜2.81~560 (97%) | ＜0.18~72.4 (86%) | ＜1.2~279 (38%) | ＜8.37~956 (30%) |  | ＜31~240 (19%) |  |  | [59] |
| Indoor parking lot dust | Guangzhou, China | 2021 | 60 | ng/g | ＜75.5~10259 (98%) |  | ＜120~4744 (85%) |  | 177~4116 (100%) |  |  | 337~21235 (100%) | ＜0.4~28.9 (75%) |  | [60] |
| Parking lot dust | Guangzhou, China | 2022~2023 | 29 | ng/g |  | ＜2.22~426 (86%) | 13~240 (100%) | 1.41~60.8 (100%) | 31~1300 (100%) | ＜8.37~214 (52%) |  | ＜31~51.5 (7%) |  |  | [59] |
| Vehicle repair plant dust | Guangzhou, China | 2022~2023 | 24 | ng/g |  | ＜2.22~1760 (50%) | 11.8~36.5 (100%) | 1.06~6.24 (100%) | 39~191 (100%) | ＜8.37~67.7 (33%) |  | ＜31~68 (17%) |  |  | [59] |
| E-waste dismantling industrial park dust | Qingyuan, China | 2016~2017 | 43 | ng/g | 32.8~990 (409, 100%) | 34.3~793 (199, 100%) | 348~2960 (1160, 100%) | 16.5~142 (61.1, 100%) | ＜3~260 (32, 84%) | ＜2~114 (10.7, 60%) |  |  |  |  | [64] |
| Soil | Milan, Italy | — | 5 | ng/g |  |  | ＜MDL~361 (20%) |  |  |  |  |  |  |  | [65] |
| Soil (rubberized playground) | Albany, New York, USA | 2022 | 3 | ng/g dw | 38~86 (62, 100%) |  | 4.6~17 (9.7, 100%) | 1.4~2.8 (2.2, 100%) | 17~110 (60, 100%) |  |  |  |  |  | [66] |
| Soil (residential area) | Albany, New York, USA | 2022 | 4 | ng/g dw | 40~450 (160, 100%) |  | 2.9~82 (25, 100%) | ＜0.2~11 (5.5, 50%) | 2.4~65 (20, 100%) |  |  |  |  |  | [66] |
| Soil (parking lot) | Albany, New York, USA | 2022 | 10 | ng/g dw | 45~960 (310, 100%) |  | 4.7~1000 (160, 100%) | 0.9~53 (9.1, 100%) | 5.3~690 (140, 100%) |  |  |  |  |  | [66] |
| Soil (garden) | Albany, New York, USA | 2022 | 3 | ng/g dw | 19~41 (32, 100%) |  | 2.1~3.5 (2.8, 100%) |  | 1.4~1.5 (1.5, 100%) |  |  |  |  |  | [66] |
| Soil (rubberized playground) | New York City, USA | 2022 | 4 | ng/g dw | 40~1800 (620, 100%) |  | 5.2~320 (160, 100%) | 0.7~58 (18, 100%) | 4.8~880 (240, 100%) |  |  |  |  |  | [66] |
| Soil (residential area) | New York City, USA | 2022 | 15 | ng/g dw | 20~1100 (250, 100%) |  | 3.2~51 (17, 100%) | ＜0.2~8.4 (2, 87%) | 2.9~69 (19, 100%) |  |  |  |  |  | [66] |
| Soil (garden/park) | New York City, USA | 2022 | 13 | ng/g dw | 28~360 (100, 100%) |  | 2.8~150 (19, 100%) | ＜0.2~0.7 (0.4, 62%) | ＜0.8~17 (5.4, 92%) |  |  |  |  |  | [66] |
| Soil (roadside) | Four cities, northeastern USA | 2022 | 47 | ng/g dw | 13~2700 (370, 100%) |  | 2.1~270 (56, 100%) | ＜0.2~24 (4.5, 92%) | ＜0.8~350 (51, 98%) |  |  |  |  |  | [66] |
| Soil (roadside) | Tianjin, China | 2016 | 10 | ng/g | diameter ＜75 μm: ＜LOQ~20 (55%) ＜10 μm: 5090~66040 (100%) ＜2.5 μm: ＜LOQ~31060 (91%) | diameter ＜75 μm: ＜LOQ~10 (64%) ＜10 μm: 0.00~80 (100%) ＜2.5 μm: 600~4500 (100%) | diameter ＜75 μm: 20~150 (100%) ＜10 μm: 2070~22980 (100%) ＜2.5 μm: 1370~25040 (100%) | diameter ＜75 μm: 10~80 (100%) ＜10 μm: ＜LOQ~1790 (73%) ＜2.5 μm: ＜LOQ~2600 (91%) | diameter ＜75 μm: ＜LOQ~10 (98%) ＜10 μm: 330~4750 (100%) ＜2.5 μm: 200~2560 (100%) |  |  |  |  |  | [58] |
| Road snow | Leipzig, Germany | 2021 | 20 | ng/L | 134.76~1845.34 (100%) |  | 286~2421 (100%) |  | n.d.~658.14 (95%) |  |  |  | n.d.~23.08 (75%) |  | [67] |
| Aquatic organism | Pearl River estuary, China | 2021 | Shrimp: 21 Sea cucumber: 5 Snail: 12 Fish: 56 | ng/g ww | Shrimp: n.d.~41 Sea cucumber: 37 Snail: ＜32~84 Fish: n.d.~80 |  | Shrimp: n.d.~10 Sea cucumber: ＜9.9 Snail: n.d. Fish: n.d.~12 |  | Shrimp: ＜0.68~4.3 Sea cucumber: 7.2 Snail: 1.1~5.2 Fish: ＜0.68~3.4 |  |  |  |  |  | [2] |
| Female *Somateria mollissima* plasma | Bengtskär colony, outer Archipelago Sea, Finland | 2021 | 18 | ng/g ww |  | Early incubation: 0.58 (5.5%) Late incubation: 0.34~5.96 (11.1%) | Early incubation: 1.57 (5.5%) Late incubation: n.d. |  |  |  | Early incubation: 0.1 (5.5%) Late incubation: n.d. |  |  |  | [68] |
| Marine mollusk | Nine coastal cities, China | 2006~2014 | 166 | ng/g dw | 132~13400 (595, 100%) |  | ＜2.44~512 (20.1, 89.2%) | ＜0.0751~15.2 (0.165, 71.1%) | 5.18~48.9 (14.2, 100%) | ＜2.06~114 (24.2, 99.4%) | 9.1~829 (67.1, 100%) |  |  | 229~13800 (778, 100%) | [69] |
| Seafood | Canary Islands, Spain | — | — | ng/g ww | 1.5~8.3 (4.6, 100%) |  |  |  | n.d.~2.2 (1.4, 75%) |  |  |  |  | 1.5~8.3 (5.3, 44%) | [70] |
| Seafood | Tarragona, Spain | 2019~2020 | 40 | ng/g ww | n.d.~84.9 (15.4, 73%) |  | n.d.~96.08 (8.95, 45%) | n.d.~9.9 (4.91, 40%) | n.d.~19.3 (3.37, 40%) |  |  |  |  |  | [71] |
| Farmed fish | Tarragona, Spain | — | 39 | ng/g dw | Turbot: n.d.~30 (2.5, 8%) Sea bass: n.d.~40 (3.3, 8%) Sea bream: n.d.~61 (7.7, 15%) |  | Turbot: n.d.~40 (11.7, 33%) Sea bass: n.d.~38 (6, 17%) Sea bream: n.d.~53 (17.3, 46%) | Turbot: n.d.~74 (13.9, 33%) Sea bass: n.d.~17 (6.1, 42%) Sea bream: n.d.~21 (6.1, 38%) | Turbot: n.d.~14 (3.7, 42%) Sea bass: n.d.~11 (2.6, 33%) Sea bream: n.d.~9 (1.7, 46%) |  |  | Turbot: n.d. Sea bass: n.d. Sea bream: n.d.~8 (0.7, 15%) |  |  | [72] |
| Wild fish | Tarragona, Spain | — | 35 | ng/g dw | Turbot: n.d.~83 (7.5, 9%) Sea bass: n.d.~21 (1.8, 8%) Sea bream: n.d.~33 (5.8, 25%) |  | Turbot: n.d.~41 (14, 36%) Sea bass: n.d.~234 (25.9, 25%) Sea bream: n.d.~56 (10.5, 25%) | Turbot: n.d.~46 (10.6, 27%) Sea bass: n.d.~127 (15.8, 42%) Sea bream: n.d.~37 (7.2, 33%) | Turbot: n.d.~9 (3.1, 45%) Sea bass: n.d.~11 (2.1, 25%) Sea bream: n.d.~12 (2.7, 50%) |  |  | Turbot: n.d. Sea bass: n.d. Sea bream: n.d.~1 (0.1, 17%) |  |  | [72] |
| Fish | Chung-Li city, Taiwan, China | — | 4 | ng/g dw |  |  | 15.4~26.1 (100%) |  |  |  |  |  |  |  | [73] |
| Leafy vegetable | Four countries | 2023 | 28 | ng/g dw | ＜8.3~238 (42.9%) |  | 665 (3.6%) |  |  |  |  |  |  |  | [74] |

^a^ The occurrence of BTs in the table was presented in the form of “concentration range (mean concentration or geometric mean concentration, detection frequency)”. If the references did not provide one or more of “concentration rang, mean concentration or geometric mean concentration, detection frequency”, then the missing content would not be displayed in the table. Blank cells indicate that the compound was not examined or that no relevant information was provided in the references. n.d., not detected; dw, dry weight; ww, wet weight; LOQ, limit of quantitation; LOD, limit of detection; MDL, method detection limit; WWTP, wastewater treatment plant; TSP, total suspended particulates.

Table S2. Occurrence of BTs in different human matrices ^a^.

| **Sample** | **Region** | **Date** | **N** | **Unit** | **BTH** | **MBT** | **2-OH-BTH** | **2-ABTH** | **2-Me-S-BTH** | **2-Mo-BTH** | **Total** | **Ref** |
| --- | --- | --- | --- | --- | --- | --- | --- | --- | --- | --- | --- | --- |
| Urine (common population) | Athens, Greece | 2012 | Male: 50 Female: 50 | μg/L | Male: ＜5~9.78 (5.36, 32%) Female: ＜5~8.92 (4.84, 26%) |  | Male: ＜2.5 (1.77, 8%) Female: ＜2.5~9.16 (2.45, 22%) | Male: ＜0.2~0.36 (0.18, 10%) Female: ＜0.2~0.29 (0.21, 6%) |  |  |  | [75] |
| Urine (common population) | Two cities, Japan | 2010~2011 | 36 | μg/L | ＜5~181 (22.7, 55.5%) |  | ＜2.5~3 (2.1, 16.6%) | 0.3~1.9 (0.7, 19.4%) |  | ＜0.5 (2.7%) | 0.4~184 (9.5, 69.4%) | [76] |
| Urine (common population) | Three cities, Korea | 2010~2011 | 49 | μg/L | ＜5~17 (6.4, 48.9%) |  | ＜2.5~5.4 (2.8, 8.1%) | 0.3~2.7 (1.2, 6.1%) |  |  | 0.3~17 (5.9, 53%) | [76] |
| Urine (common population) | Mettupalayam, India | 2010~2011 | 46 | μg/L | ＜5~23.2 (5.4, 41.3%) |  | 3.8~14.7 (7.5, 4.3%) | 0.8~2.1 (1.3, 6.5%) |  | ＜0.5~2.5 (0.6, 13%) | 0.6~23.2 (4.5, 42.8%) | [76] |
| Urine (common population) | Hanoi, Vietnam | 2010~2011 | 25 | μg/L | ＜5~113 (10.4, 100%) |  | ＜2.5~2.8 (2.2, 16%) | 1.1 (4%) |  |  | 3.6~113 (10.7, 100%) | [76] |
| Urine (common population) | Three cities, China | 2010~2011 | 51 | μg/L | ＜5~40.7 (7.6, 37.2%) |  | ＜2.5~9.5 (2.7, 15.6%) |  |  | ＜0.5~1.7 (0.8, 3.9%) | 0.4~40 (5.6, 50.9%) | [76] |
| Urine (common population) | Albany, New York, USA | 2010~2011 | 25 | μg/L | ＜5~8.7 (5.7, 24%) |  | ＜2.5 (8%) | ＜0.2~2.3 (1, 12%) |  |  | 0.2~8.7 (2.8, 44%) | [76] |
| Urine (common population) | Athens, Greece | 2012 | 100 | μg/L | ＜5~9.8 (5.1, 29%) |  | ＜2.5~9.2 (2.3, 15%) | ＜0.2~0.4 (0.2, 8%) |  | 0.7~3.8 (1.6, 2%) | 0.1~12 (3.4, 40%) | [76] |
| Urine (common population) | Taiwan, China |  | Male: 10 Female: 10 | μg/L | Male: n.d. Female: n.d. |  | Male: n.d. Female: n.d.~＜4 | Male: n.d.~21.1 Female: n.d.~24.1 | Male: n.d. Female: n.d. |  |  | [77] |
| Urine (common population) | Germany | 2015~2017 | 516 | μg/L |  | ＜1~43.5 (1.018, 50%) |  |  |  |  |  | [78] |
| Urine (common population) | Taizhou, China | 2024 | 197 | μg/L |  | ＜0.063~22 (2.2, 89%) |  |  | ＜0.061~4.2 (0.81, 76%) |  |  | [79] |
| Urine (common population) | Czech | 2019 | 165 | μg/L |  |  | ＜0.38~21 (2.52, 83%) | ＜0.07~1.82 (＜0.07, 3%) |  |  |  | [80] |
| Urine (common population) | Australia | 2012~2023 | Male: 84 pools Female: 84 pools | μg/L | Male: ＜0.95~9.3 (4.3, 73%) Female: ＜0.95~9.6 (4.7, 73%) |  | Male: ＜1.2~31 (12, 80%) Female: ＜1.2~34 (12, 76%) | Male: ＜0.083~1.5 (58%) Female: ＜0.083~1.4 (0.19, 64%) | Male: ＜0.0086~0.11 (1.2%) Female: ＜0.0086~0.3 (6%) |  |  | [81] |
| Urine (common population) | Quzhou, China | 2019~2020 | 212 | μg/g creatinine | (2.29, 85%) |  | (0.78, 68%) | (0.18, 63%) | (0.65, 86%) |  |  | [82] |
| Urine (lung cancer patients) | Quzhou, China | 2019~2020 | 212 | μg/g creatinine | (2.38, 89%) |  | (0.81, 76%) | (0.1, 65%) | (0.58, 75%) |  |  | [82] |
| Urine (sawmill workers) | Finland |  | 26 | μmol/L |  | ＜0.12~0.15 |  |  |  |  |  | [83] |
| Urine (pregnant woman) | Tianjin, China | 2015 | 83 | μg/L | 0.016~3.91 (1.13, 100%) |  |  |  |  |  |  | [84] |
| Serum (common population) | Zhuhai, China | 2022 | 143 | μg/L | ＜0.05~28 (0.45, 58%) |  |  | ＜0.02~2.61 (0.63, 96%) |  |  |  | [85] |
| Serum (non-alcoholic fatty liver disease patients) | Zhuhai, China | 2022 | 138 | μg/L | ＜0.05~259 (0.76, 79%) |  |  | ＜0.02~2.81 (0.43, 96%) |  |  |  | [85] |
| Amniotic fluid (pregnant woman) | Tianjin, China | 2015 | 79 | μg/L | ＜LOD~3.01 (0.63, 84.8%) |  | ＜LOD~0.52 (0.3, 32.9%) |  |  |  |  | [84] |
| Breast milk (common population) | Seven provinces, China |  | 20 | ng/g lw | (102, 90%) |  |  |  |  |  |  | [86] |
| Thrombogenic coronary plaques (heart disease patients’ corpse) | New Orleans, Louisiana, USA |  | 2 | ng/g | 10 (50%) |  |  |  |  |  |  | [87] |
| Adipose tissue (common population) | New York City, New York, USA | 2003~2004 | 20 | ng/g | ＜10~20.2 (30%) |  | ＜4~62.5 (5.5, 55%) |  | ＜2~8.18 (5%) |  |  | [88] |
| Exhaled breath condensate (common population) | Zurich, Switzerland |  | 10 | μg/L | 30~43 (37, 100%) | ＜LOD~15 (6.7, 60%) | 20~26 (23, 100%) | ＜LOD~27 (11, 90%) | 15~42 (26, 100%) | 4~14 (7, 100%) |  | [89] |
| Semen (common population) | Spain | 2021 | 10 | μg/L |  | n.d.~0.83 (0.08, 20%) |  |  |  |  |  | [90] |

^a^ The occurrence of BTs in the table was presented in the form of “concentration rang (mean concentration or geometric mean concentration, detection frequency)”. If the references did not provide one or more of “concentration rang, mean concentration or geometric mean concentration, detection frequency”, then the missing content would not be displayed in the table. Blank cells indicate that the compound was not examined or that no relevant information was provided in the references. n.d., not detected; lw, lipid weight; LOD, limit of detection.

Table S3. Specific types of BTs positive targets.

| **Compound** | **Predicted positive targets (TargetNet, SwissTargetPrediction and ChEMBL database)** | **Positive targets (Tox21 database)** |
| --- | --- | --- |
| BTH | AHR、ALDH1A1、ATP4A、CA13、CA5A、CA6、CASP9、CES1、CES2、DUSP3、DYRK1A、GMNN、GRM4、HSD17B10、IDO1、IKBKB、MGLL、MIF、NOS1、NOS2、NOS3、NR2E3、PDE4A、PDE4B、PDE4C、PDE4D、PPO2、RELA、RORA、TAAR1、TARDBP、TLR9、TRPA1 | AHR |
| 2-Me-BTH | AHR、AKR1B1、ALOX5、ALPL、APOBEC3G、APP、ATP4A、BCL2A1、CA13、CA5A、CA5B、CA6、CES1、CYP1A2、DUSP3、DYRK1A、GRM4、GRM5、HSD17B2、IDO1、ILK、LEF、MGLL、MIF、NOS1、NOS3、NR2E3、PLIN5、PPO2、PTGS1、PTPN7、RELA、RORA、TLR9 | CYP2C19、ESRRA |
| 2-ABTH | AHR、ALPL、AOC3、APP、ATAD5、ATP4A、BCL2A1、CA13、CA4、CA5A、CA5B、CA6、CA7、DAO、DHFR、DYRK1A、GRM4、HRH4、IDO1、LIMK1、MAPK1、MAPK3、MIF、NCF1、NOS1、NOS3、NPY5R、PI4KA、PI4KB、PIK3CB、PIK3CD、PPO2、RELA、ROCK1、RORA、RORC、SLC6A2、TLR9 | AHR、CYP1A2、CYP2D6、ESRRA、HDAC9、NR1I3 |
| MBT | AHR、ADRA2C、ALDH1A1、ALOX15、ALOX15B、ALPL、APEX1、APOBEC3G、BCL2A1、CA4、CA5A、CA5B、CA6、CASP9、CES2、CYP3A4、DRD1、DRD2、DRD4、DRD5、DYRK1A、EHMT2、HDAC6、HPGD、HTR1B、HTR2C、HTR5A、HTR6、IDO1、KAT2A、KDM4E、MGLL、MIF、NFE2L2、NR2E3、PDK1、PPARG、PPO2、PSMB5、PSMB8、RELA、RORA、RXRA、SLCO1B1、SLCO1B3、TDP1、THRB、TLR9、UGT2B7、VDR | AHR、CYP1A2、CYP2C9、CYP2C19、CYP2D6、ESRRA、GLI3、HSPB1、JUN、NFE2L2、NR1I2、NR1I3、PGR、PPARG、RARA |
| 2-SCN-Me-S-BTH | AHR、ABCC8、ADORA1、ALPL、APOBEC3G、ATP4A、BCL2A1、CA13、CACNA1H、CBFB、CCNA1、CCNA2、CCNB1、CCNB2、CCNB3、CCR4、CDK1、CDK2、CTDSP1、CTSL、DRD4、DUSP3、EGFR、EP300、ESR1、FAAH、GRM5、KCNJ11、KDR、LRRK2、MAOA、MAOB、MAPK1、NFKB1、NOS1、NOS3、NR1H4、NR3C1、PDE4A、PDE7A、PIM1、PPARD、PPARG、RELA、RORA、RXRA、THRB、TLR9、TP53、VDR | AR、ATAD5、CASP3、CASP7、CYP1A2、CYP2C9、CYP2C19、CYP2D6、CYP19A1、ESRRA、GLI3、HDAC9、HSPB1、JUN、NFE2L2、NR1H4、NR1I2、NR1I3、NR3C1、PPARG、RARA、Rorc、SMAD2、SMAD3、Thrb、TP53、TRHR、TSHR |
| 2-OH-BTH | AHR、AKR1B1、ALOX5、ALPL、BCL2A1、CA13、CA4、CA5A、CA5B、CA6、CA7、CASP9、CES1、CES2、DYRK1A、EHMT2、GNAS、HTR5A、HTR6、IDO1、MIF、NR2E3、PPO2、QPCT、RAPGEF3、RELA、RORA、SENP8 |  |
| 2-Me-S-BTH | AHR、AKR1B1、ALOX5、ALPL、APOBEC3G、ATP4A、BCL2A1、CA13、CA4、CA5A、CA5B、CES1、CYP1A2、DRD4、GRM4、GRM5、HNF4A、HTR1A、HTR2A、HTR7、MAOA、MGLL、MIF、NOS2、NOS3、NR2E3、PPO2、PTGS1、RELA、RORA、TLR9、TUBA1A |  |
| 2-Cl-BTH | AHR、AKR1B1、ALOX5、ALPL、APOBEC3G、APP、ATP4A、BCL2A1、CA13、CA5A、CA5B、CA6、CACNA1H、CES1、CYP1A2、DRD4、DUSP3、DYRK1A、GRM4、HSD17B2、LEF、MAOA、MAOB、MGLL、MIF、NOS1、NOS3、NR2E3、PTGS1、PTGS2、PTPN7、RELA、RORA、TLR9 |  |
| 2-Mo-BTH | AHR、ADRA1A、ADRA2B、ADRA2C、APH1A、APH1B、APOBEC3G、APP、AR、ATAD5、ATM、BACE1、BCL2A1、BRCA1、BRD3、CA13、CA14、CA3、CA4、CA5A、CA5B、CA6、CA7、CACNA1H、CBX1、CCKBR、CCNB1、CCNB2、CCNB3、CCR5、CDK1、CHRM1、CHRM2、CHRM3、CHRM4、CHRM5、CHRNA4、CSNK1D、CYP19A1、CYP1A1、CYP1A2、CYP1B1、DRD4、DUSP3、ECE1、EDNRA、EGFR、EPHB4、GABRA1、GABRA5、GABRB3、GABRG2、GLP1R、GSK3B、HRH3、HSD11B1、HTR1A、HTR6、IRAK4、JAK1、JAK2、KCNMA1、KDR、LIPE、MAOA、MAPK10、MCHR1、MKNK1、MPO、MTOR、NCSTN、NOS2、NPC1、NQO1、P2RX7、PDE10A、PDE4D、PDE5A、PDE9A、PDGFRB、PIK3C2B、PIK3CA、PIK3CB、PIK3CD、PIK3CG、PIK3R1、PIM1、PIM3、PLA2G7、PLK1、PLK3、PRKCZ、PRKDC、PSEN1、PSEN2、PSEN2、PSENEN、PTPN1、RAB9A、RAPGEF3、RELA、ROCK1、RORA、RPS6KB1、SCD1、SCN9A、SLC6A3、SLC6A4、SMN1、SMN2、TLR4、TLR9、TNKS2、TYK2、UTS2R |  |

**References**

1. R. Zhang, S. Zhao, X. Liu, L. Tian, Y. Mo, X. Yi, et al., Aquatic environmental fates and risks of benzotriazoles, benzothiazoles, and p-phenylenediamines in a catchment providing water to a megacity of China, Environ. Res. 216 (2023) 114721.
2. L.N. Wei, N.N. Wu, R. Xu, S. Liu, H.X. Li, L. Lin, et al., First evidence of the bioaccumulation and trophic transfer of tire additives and their transformation products in an estuarine food web, Environ. Sci. Technol. 58 (2024) 6370-6380.
3. L.X. Hu, Y.X. Cheng, D. Wu, L. Fan, J.H. Zhao, Q. Xiong, et al., Continuous input of organic ultraviolet filters and benzothiazoles threatens the surface water and sediment of two major rivers in the Pearl River Basin, Sci. Total Environ. 798 (2021) 149299.
4. X. Han, Z. Xie, Y. Tian, W. Yan, L. Miao, L. Zhang, et al., Spatial and seasonal variations of organic corrosion inhibitors in the Pearl River, South China: Contributions of sewage discharge and urban rainfall runoff, Environ. Pollut. 262 (2020) 114321.
5. H.Y. Zhang, Y.H. Liu, L.N. Wei, R.Q. Zhu, J.L. Zhao, S. Liu, et al., Unveiling spatiotemporal distribution, partitioning, and transport mechanisms of tire additives and their transformation products in a highly urbanized estuarine region, Sci. Total Environ. 954 (2024) 176804.
6. H.Y. Zhang, Z. Huang, Y.H. Liu, L.X. Hu, L.Y. He, Y.S. Liu, et al., Occurrence and risks of 23 tire additives and their transformation products in an urban water system, Environ. Int. 171 (2023) 107715.
7. C. Rauert, S. Vardy, B. Daniell, N. Charlton, K.V. Thomas, Tyre additive chemicals, tyre road wear particles and high production polymers in surface water at 5 urban centres in Queensland, Australia, Sci. Total Environ. 852 (2022) 158468.
8. E. Fries, T. Gocht, J. Klasmeier, Occurrence and distribution of benzothiazole in the Schwarzbach watershed (Germany), J. Environ. Monit. 13 (2011) 2838-2843.
9. P. Herrero, F. Borrull, E. Pocurull, R.M. Marce, Efficient tandem solid-phase extraction and liquid chromatography-triple quadrupole mass spectrometry method to determine polar benzotriazole, benzothiazole and benzenesulfonamide contaminants in environmental water samples, J. Chromatogr. A 1309 (2013) 22-32.
10. C. Rauert, N. Charlton, E.D. Okoffo, R.S. Stanton, A.R. Agua, M.C. Pirrung, et al., Concentrations of tire additive chemicals and tire road wear particles in an Australian urban tributary, Environ. Sci. Technol. 56 (2022) 2421-2431.
11. L. Dsikowitzky, I. Nordhaus, C.H. Sujatha, P.S. Akhil, K. Soman, J. Schwarzbauer, A combined chemical and biological assessment of industrial contamination in an estuarine system in Kerala, India, Sci. Total Environ. 485 (2014) 348-362.
12. Y.T. Ao, Y.C. Chen, W.H. Ding, Deep eutectic solvent-based ultrasound-assisted emulsification microextraction for the rapid determination of benzotriazole and benzothiazole derivatives in surface water samples, J. Hazard. Mater. 401 (2021) 123383.
13. S.M.d. Ochs, T.M. Souza, R.d.L. Sobrinho, R.B. de Oliveira, M.C. Bernardes, A.D.P. Netto, Simultaneous evaluation of benzotriazoles, benzothiazoles and benzenesulfonamides in water samples from the impacted urban Jacarepagua Lagoon System (Rio de Janeiro, Brazil) by liquid chromatography coupled to electrospray tandem mass spectrometry, Sci. Total Environ. 858 (2023) 160033.
14. L. Kong, K. Kadokami, S. Wang, D. Hanh Thi, C. Hong Thi Cam, Monitoring of 1300 organic micro-pollutants in surface waters from Tianjin, North China, Chemosphere 122 (2015) 125-130.
15. M.L. Zhao, Y. Chen, G.P. Yang, R. Chen, Simultaneous determination of benzothiazoles, benzotriazoles, and benzotriazole UV absorbers by solid-phase extraction-gas chromatography-mass spectrometry, Environ. Sci. Pollut. Res. 30 (2023) 45315-45330.
16. M.L. Zhao, X. Ji, J. Zhang, G.P. Yang, Spatiotemporal variation, partitioning, and ecological risk assessment of benzothiazoles, benzotriazoles, and benzotriazole UV absorbers in the Yangtze River Estuary and its adjacent area, J. Hazard. Mater. 465 (2024) 133337.
17. M.L. Zhao, J. Fu, X. Ji, J. Zhang, Z. He, G.P. Yang, Comprehensive analysis of benzothiazoles (BTHs), benzotriazoles (BTRs), and benzotriazole ultraviolet absorbers (BUVs) in the western South China Sea: Spatial distributions, migration tendencies and ecotoxicological relevance, Water Res. 266 (2024) 122372.
18. A. Munteanu, M. Bortolini, M. Feltracco, A. Alterio, W.R.L. Cairns, C. Turetta, et al., Contamination by benzothiazoles in the Arctic: First evidence in the seawater of the Greenland Sea, Environ. Pollut. 371 (2025) 125943.
19. C.M. Reddy, J.G. Quinn, Environmental chemistry of benzothiazoles derived from rubber, Environ. Sci. Technol. 31 (1997) 2847-2853.
20. H.E. Fuchte, N. Beck, E. Bieg, V.J. Bayer, C. Achten, M. Krauss, et al., A look down the drain: Identification of dissolved and particle bound organic pollutants in urban runoff waters and sediments, Environ. Pollut. 302 (2022) 119047.
21. M. Grung, S. Meland, A. Ruus, S. Ranneklev, E. Fjeld, A. Kringstad, et al., Occurrence and trophic transport of organic compounds in sedimentation ponds for road runoff, Sci. Total Environ. 751 (2021) 141808.
22. S. Meland, G.M. Granheim, J.T. Rundberget, E. Rodland, Screening of tire-derived chemicals and tire wear particles in a road tunnel wash water treatment basin, Environ. Sci. Technol. Lett. 11 (2023) 35-40.
23. H.G. Ni, F.H. Lu, X.L. Luo, H.Y. Tian, E.Y. Zeng, Occurrence, phase distribution, and mass loadings of benzothiazoles in riverine runoff of the Pearl River Delta, China, Environ. Sci. Technol. 42 (2008) 1892-1897.
24. Y.H. Liu, Y.X. Mei, X.N. Liang, Z.Y. Ge, Z. Huang, H.Y. Zhang, et al., Small-intensity rainfall triggers greater contamination of rubber-derived chemicals in road stormwater runoff from various functional areas in megalopolis cities, Environ. Sci. Technol. 58 (2024) 13056-13064.
25. W. Wang, S. Park, B.g. Choi, J.E. Oh, Occurrence and removal of benzotriazole and benzothiazole in drinking water treatment plants, Environ. Pollut. 316 (2023) 120563.
26. W. Sim, K.E. Muambo, J. Choi, S. Park, J.E. Oh, Occurrence, distribution, and prioritization of unregulated emerging contaminants including battery-related chemicals in drinking water systems across South Korea, Sci. Total Environ. 967 (2025) 178799.
27. L. Wang, J. Zhang, H. Sun, Q. Zhou, Widespread occurrence of benzotriazoles and benzothiazoles in tap water: Influencing factors and contribution to human exposure, Environ. Sci. Technol. 50 (2016) 2709-2717.
28. C.J. Hsu, W.H. Ding, Determination of benzotriazole and benzothiazole derivatives in tea beverages by deep eutectic solvent-based ultrasound-assisted liquid-phase microextraction and ultrahigh-performance liquid chromatography-high resolution mass spectrometry, Food Chem. 368 (2022) 130798.
29. V. Bellavia, M. Natangelo, R. Fanelli, D. Rotilio, Analysis of benzothiazole in Italian wines using headspace solid-phase microextraction and gas chromatography-mass spectrometry, J. Agric. Food Chem. 48 (2000) 1239-1242.
30. B. Fedrizzi, F. Magno, D. Badocco, G. Nicolini, G. Versini, Aging effects and grape variety dependence on the content of sulfur volatiles in wine, J. Agric. Food Chem. 55 (2007) 10880-10887.
31. B. Fedrizzi, F. Magno, F. Finato, G. Versini, Variation of some fermentative sulfur compounds in Italian "Millesime" classic sparkling wines during aging and storage on lees, J. Agric. Food Chem. 58 (2010) 9716-9722.
32. A.G. Asimakopoulos, A. Ajibola, K. Kannan, N.S. Thomaidis, Occurrence and removal efficiencies of benzotriazoles and benzothiazoles in a wastewater treatment plant in Greece, Sci. Total Environ. 452 (2013) 163-171.
33. A. Kloepfer, M. Jekel, T. Reemtsma, Occurrence, sources, and fate of benzothiazoles in municipal wastewater treatment plants, Environ. Sci. Technol. 39 (2005) 3792-3798.
34. I. Carpinteiro, B. Abuin, M. Ramil, I. Rodriguez, R. Cela, Simultaneous determination of benzotriazole and benzothiazole derivatives in aqueous matrices by mixed-mode solid-phase extraction followed by liquid chromatography-tandem mass spectrometry, Anal. Bioanal. Chem. 402 (2012) 2471-2478.
35. T. Reemtsma, Determination of 2-substituted benzothiazoles of industrial use from water by liquid chromatography/electrospray ionization tandem mass spectrometry, Rapid Commun. Mass Spectrom. 14 (2000) 1612-1618.
36. J. Wang, Z. Tian, Y. Huo, M. Yang, X. Zheng, Y. Zhang, Monitoring of 943 organic micropollutants in wastewater from municipal wastewater treatment plants with secondary and advanced treatment processes, J. Environ. Sci. 67 (2018) 309-317.
37. R. Karthikraj, K. Kannan, Mass loading and removal of benzotriazoles, benzothiazoles, benzophenones, and bisphenols in Indian sewage treatment plants, Chemosphere 181 (2017) 216-223.
38. Z.M. Li, K. Kannan, Mass loading, removal, and emission of 1,3-diphenylguanidine, benzotriazole, benzothiazole, N-(1,3-dimethylbutyl)-N′-phenyl-p-phenylenediamine, and their derivatives in a wastewater treatment plant in New York State, USA, ACS ES&T Water 4 (2024) 2721-2730.
39. A.S. Stasinakis, N.S. Thomaidis, O.S. Arvaniti, A.G. Asimakopoulos, V.G. Samaras, A. Ajibola, et al., Contribution of primary and secondary treatment on the removal of benzothiazoles, benzotriazoles, endocrine disruptors, pharmaceuticals and perfluorinated compounds in a sewage treatment plant, Sci. Total Environ. 463 (2013) 1067-1075.
40. J. Xue, Y. Lin, D. Zhao, K. Kannan, Occurrence, removal, and fate of benzothiazoles (BTHs) and benzotriazoles (BTRs) in two wastewater treatment plants in New York State, USA, Sci. Total Environ. 949 (2024) 175090.
41. P. Herrero, F. Borrull, R.M. Marce, E. Pocurull, A pressurised hot water extraction and liquid chromatography-high resolution mass spectrometry method to determine polar benzotriazole, benzothiazole and benzenesulfonamide derivates in sewage sludge, J. Chromatogr. A 1355 (2014) 53-60.
42. A. Wick, G. Fink, T.A. Ternes, Comparison of electrospray ionization and atmospheric pressure chemical ionization for multi-residue analysis of biocides, UV-filters and benzothiazoles in aqueous matrices and activated sludge by liquid chromatography-tandem mass spectrometry, J. Chromatogr. A 1217 (2010) 2088-2103.
43. P. Herrero, F. Borrull, E. Pocurull, R.M. Marce, A quick, easy, cheap, effective, rugged and safe extraction method followed by liquid chromatography-(Orbitrap) high resolution mass spectrometry to determine benzotriazole, benzothiazole and benzenesulfonamide derivates in sewage sludge, J. Chromatogr. A 1339 (2014) 34-41.
44. J. Carlsson, F. Iadaresta, J. Eklund, R. Avagyan, C. Ostman, U. Nilsson, Suspect and non-target screening of chemicals in clothing textiles by reversed-phase liquid chromatography/hybrid quadrupole-Orbitrap mass spectrometry, Anal. Bioanal. Chem. 414 (2022) 1403-1413.
45. R. Avagyan, G. Luongo, G. Thorsen, C. Ostman, Benzothiazole, benzotriazole, and their derivates in clothing textiles-a potential source of environmental pollutants and human exposure, Environ. Sci. Pollut. Res. 22 (2015) 5842-5849.
46. R. Avagyan, I. Sadiktsis, G. Thorsen, C. Ostman, R. Westerholm, Determination of benzothiazole and benzotriazole derivates in tire and clothing textile samples by high performance liquid chromatography-electrospray ionization tandem mass spectrometry, J. Chromatogr. A 1307 (2013) 119-125.
47. W. Liu, J. Xue, K. Kannan, Occurrence of and exposure to benzothiazoles and benzotriazoles from textiles and infant clothing, Sci. Total Environ. 592 (2017) 91-96.
48. R. Garcia-Garcinuno, L. Vallecillos, R.M. Marce, F. Borrull, Occurrence of high production volume chemicals and polycyclic aromatic hydrocarbons in urban sites close to industrial areas. Human exposure and risk assessment, Chemosphere 351 (2024) 141167.
49. L. Tian, S. Zhao, R. Zhang, S. Lv, D. Chen, J. Li, et al., Tire wear chemicals in the urban atmosphere: Significant contributions of tire wear particles to PM_2.5_, Environ. Sci. Technol. 58 (2024) 16952-16961.
50. V. Kuntz, D. Zahn, T. Reemtsma, Quantification and occurrence of 39 tire-related chemicals in urban and rural aerosol from Saxony, Germany, Environ. Int. 194 (2024) 109189.
51. A. Maceira, R. Maria Marce, F. Borrull, Occurrence of benzothiazole, benzotriazole and benzenesulfonamide derivates in outdoor air particulate matter samples and human exposure assessment, Chemosphere 193 (2018) 557-566.
52. X. Liao, T. Zou, M. Chen, Y. Song, C. Yang, B. Qiu, et al., Contamination profiles and health impact of benzothiazole and its derivatives in PM_2.5_ in typical Chinese cities, Sci. Total Environ. 755 (2021) 142617.
53. E. Favaro, G. Mazzi, E. Barbaro, M. Masiol, A. Alterio, A. Gambaro, et al., Occurrence of tyre-derived particles in size-segregated aerosol in the urban area of Venice, Atmos. Environ. 337 (2024) 120784.
54. Y.J. Wan, J.C. Xue, K. Kannan, Benzothiazoles in indoor air from Albany, New York, USA, and its implications for inhalation exposure, J. Hazard. Mater. 311 (2016) 37-42.
55. M. Feltracco, G. Mazzi, E. Barbaro, E. Gregoris, M. Bortolini, C. Barbante, et al., Insights into size-segregated distribution of benzothiazoles in indoor aerosol from office environments, Environ. Sci. - Atmospheres 4 (2024) 571-577.
56. W. Wang, J. Zhang, G. Huang, I. Pryjomska-Ray, D.A. Volmer, Z. Cai, Tire-additive chemicals and their derivatives in urban road dust: Spatial distributions, exposures, and associations with tire and road wear particles, J. Hazard. Mater. 490 (2025) 137749.
57. J. Asheim, K. Vike-Jonas, S.V. Gonzalez, S. Lierhagen, V. Venkatraman, I.L.S. Veivåg, et al., Benzotriazoles, benzothiazoles and trace elements in an urban road setting in Trondheim, Norway: Re-visiting the chemical markers of traffic pollution, Sci. Total Environ. 649 (2019) 703-711.
58. J. Zhang, X.F. Zhang, L. Wu, T. Wang, J.B. Zhao, Y.J. Zhang, et al., Occurrence of benzothiazole and its derivates in tire wear, road dust, and roadside soil, Chemosphere 201 (2018) 310-317.
59. J. Ge, X. Hou, L. Liu, Q. Deng, B. Du, L. Zeng, Comprehensive identification and ubiquitous occurrence of eight classes of rubber-derived vulcanization accelerators in urban dusts, Environ. Sci. Technol. 58 (2024) 5117-5128.
60. C. Deng, J. Huang, Y. Qi, D. Chen, W. Huang, Distribution patterns of rubber tire-related chemicals with particle size in road and indoor parking lot dust, Sci. Total Environ. 844 (2022) 157144.
61. M. Nunez, N. Fontanals, F. Borrull, R.M. Marcie, Multiresidue analytical method for high production volume chemicals in dust samples, occurrence and human exposure assessment, Chemosphere 301 (2022) 134639.
62. L. Wang, A.G. Asimakopoulos, H.B. Moon, H. Nakata, K. Kannan, Benzotriazole, benzothiazole, and benzophenone compounds in indoor dust from the United States and East Asian countries, Environ. Sci. Technol. 47 (2013) 4752-4759.
63. Q. Zhu, C. Liao, G. Jiang, Occurrence of human exposure to benzothiazoles and benzotriazoles in indoor dust in Suizhou and Beijing, China, Chem. Res. Chin. Univ. 39 (2023) 508-515.
64. W. Li, J. Li, M. Deng, Y. Pan, L. Zeng, Benzotriazoles and benzothiazoles prevail in indoor dust from an E-waste dismantling area in South China: Elevated concentrations and implication for human exposure, Sci. Total Environ. 723 (2020) 137979.
65. A. Speltini, M. Sturini, F. Maraschi, A. Porta, A. Profumo, Fast low-pressurized microwave-assisted extraction of benzotriazole, benzothiazole and benezenesulfonamide compounds from soil samples, Talanta 147 (2016) 322-327.
66. Z.M. Li, V.K. Pal, P. Kannan, W.L. Li, K. Kannan, 1,3-Diphenylguanidine, benzothiazole, benzotriazole, and their derivatives in soils collected from northeastern United States, Sci. Total Environ. 887 (2023) 164110.
67. L. Maurer, E. Carmona, O. Machate, T. Schulze, M. Krauss, W. Brack, Contamination pattern and risk assessment of polar compounds in snow melt: An integrative proxy of road runoffs, Environ. Sci. Technol. 57 (2023) 4143-4152.
68. A.V. Ask, V.L.B. Jaspers, J. Zhang, A.G. Asimakopoulos, S.H. Froyland, J. Jolkkonen, et al., Contaminants of emerging concern in an endangered population of common eiders (Somateria mollissima) in the Baltic Sea, Environ. Pollut. 365 (2025) 125409.
69. J. Jia, Q. Zhu, N. Liu, C. Liao, G. Jiang, Occurrence of and human exposure to benzothiazoles and benzotriazoles in mollusks in the Bohai Sea, China, Environ. Int. 130 (2019) 104925.
70. O. Castro, S. Borrull, J. Riu, S. Gimeno-Monforte, S. Montesdeoca-Esponda, Z. Sosa-Ferrera, et al., Seafood consumption as a source of exposure to high production volume chemicals: A comparison between Catalonia and the Canary Islands, Food Chem. Toxicol. 175 (2023) 113729.
71. O. Castro, S. Borrull, F. Borrull, E. Pocurull, High production volume chemicals in the most consumed seafood species in Tarragona area (Spain): Occurrence, exposure, and risk assessment, Food Chem. Toxicol. 173 (2023) 113625.
72. S. Borrull, F. Borrull, E. Pocurull, R.M. Marce, Comparison of the presence of high production volume chemicals in farmed and wild fish highly consumed in catalonia and their risk assessment, Chemosphere 365 (2024) 143364.
73. C.H. Chen, W.H. Chung, W.H. Ding, Determination of benzotriazole and benzothiazole derivatives in marketed fish by double-vortex-ultrasonic assisted matrix solid-phase dispersion and ultrahigh-performance liquid chromatography-high resolution mass spectrometry, Food Chem. 333 (2020) 127516.
74. A. Sherman, L.E. Haemmerle, E. Ben Mordechay, B. Chefetz, T. Hueffer, T. Hofmann, Uptake of tire-derived compounds in leafy vegetables and implications for human dietary exposure, Front. Environ. Sci. 12 (2024) 1384506.
75. A.G. Asimakopoulos, A.A. Bletsou, Q. Wu, N.S. Thomaidis, K. Kannan, Determination of benzotriazoles and benzothiazoles in human urine by liquid chromatography-tandem mass spectrometry, Anal. Chem. 85 (2013) 441-448.
76. A.G. Asimakopoulos, L. Wang, N.S. Thomaidis, K. Kannan, Benzotriazoles and benzothiazoles in human urine from several countries: A perspective on occurrence, biotransformation, and human exposure, Environ. Int. 59 (2013) 274-281.
77. Y.J. Li, W.H. Ding, Determination of benzotriazole and benzothiazole derivatives in human urine by eco-friendly deep eutectic solvent-based ultrasound-assisted liquid-liquid microextraction followed by ultrahigh performance liquid chromatography quadrupole-time-of-flight mass spectrometry, Environ. Pollut. 284 (2021) 117530.
78. A. Murawski, M.I.H. Schmied-Tobies, G. Schwedler, E. Rucic, W. Gries, C. Schmidtkunz, et al., 2-Mercaptobenzothiazole in urine of children and adolescents in Germany - Human biomonitoring results of the German Environmental Survey 2014-2017 (GerES V), Int. J. Hyg. Environ. Health 228 (2020) 113540.
79. X. Wu, Y. Zhu, R. Guo, J. Huang, H. Jin, L. Zhou, 2-Mercaptobenzothiazole-derived vulcanization accelerators in urine samples from Chinese adults, Sci. Total Environ. 955 (2024) 176815.
80. N. Palesova, L. Blahova, T. Janos, K. Rihackova, A. Pindur, L. Sebejova, et al., Exposure to benzotriazoles and benzothiazoles in Czech male population and its associations with biomarkers of liver function, serum lipids and oxidative stress, Int. Arch. Occup. Environ. Health 97 (2024) 523-536.
81. D.E. Que, X. Wang, S. Nilsson, I. Zammit, D.C.G. Muir, C. Rauert, et al., Trends of benzotriazoles and benzothiazoles in Australian pooled urine samples from 2012 to 2023, Environ. Sci. Technol. 58 (2024) 19960-19969.
82. W. Mao, J. Qu, H. Liu, R. Guo, K. Liao, S. Wu, et al., Associations between urinary concentrations of benzothiazole, benzotriazole, and their derivatives and lung cancer: A nested case-control study, Environ. Res. 251 (2024) 118750.
83. A. Manninen, S. Auriola, M. Vartiainen, J. Liesivuori, T. Turunen, M. Pasanen, Determination of urinary 2-mercaptobenzothiazole (2-MBT), the main metabolite of 2-(thiocyanomethylthio)benzothiazole (TCMTB) in humans and rats, Arch. Toxicol. 70 (1996) 579-584.
84. X. Li, L. Wang, A.G. Asimakopoulos, H. Sun, Z. Zhao, J. Zhang, et al., Benzotriazoles and benzothiazoles in paired maternal urine and amniotic fluid samples from Tianjin, China, Chemosphere 199 (2018) 524-530.
85. R. Qin, B. Zhang, H. Zhu, Y. Chen, S. Song, T. Zhang, Exposure to per- and polyfluoroalkyl substances, neonicotinoid insecticides, benzotriazoles and benzothiazoles: Associations with human non-alcoholic fatty liver disease, Environ. Chem. Ecotoxicol. 6 (2024) 283-292.
86. Y. Zhang, L. Gao, Q. Ai, Y. Liu, L. Qiao, X. Cheng, et al., Screening for compounds with bioaccumulation potential in breast milk using their retention behavior in two-dimensional gas chromatography, Environ. Int. 190 (2024) 108911.
87. J.B. Ferrario, I.R. Deleon, R.E. Tracy, Evidence for toxic anthropogenic chemicals in human thrombogenic coronary plaques, Arch. Environ. Contam. Toxicol. 14 (1985) 529-534.
88. L. Wang, A.G. Asimakopoulos, K. Kannan, Accumulation of 19 environmental phenolic and xenobiotic heterocyclic aromatic compounds in human adipose tissue, Environ. Int. 78 (2015) 45-50.
89. D. Garcia-Gomez, L. Bregy, Y. Nussbaumer-Ochsner, T. Gaisl, M. Kohler, R. Zenobi, Detection and quantification of benzothiazoles in exhaled breath and exhaled breath condensate by real-time secondary electrospray ionization-high-resolution mass spectrometry and ultra-high performance liquid chromatography, Environ. Sci. Technol. 49 (2015) 12519-12524.
90. E. Sanchez-Resino, M. Marques, D. Gutierrez-Martin, E. Restrepo-Montes, M.A. Martinez, A. Salas-Huetos, et al., Exploring the occurrence of organic contaminants in human semen through an innovative LC-HRMS-Based methodology suitable for target and nontarget analysis, Environ. Sci. Technol. 57 (2023) 19236-19252.
